# Biosynthesis of the acetyl‐CoA carboxylase‐inhibiting antibiotic, andrimid in *Serratia* is regulated by Hfq and the LysR‐type transcriptional regulator, AdmX

**DOI:** 10.1111/1462-2920.13241

**Published:** 2016-05-25

**Authors:** Miguel A. Matilla, Veronika Nogellova, Bertrand Morel, Tino Krell, George P. C. Salmond

**Affiliations:** ^1^ Department of BiochemistryUniversity of CambridgeTennis Court RoadCambridgeCB2 1QWUK; ^2^ Department of Environmental ProtectionEstación Experimental del Zaidín, Consejo Superior de Investigaciones CientíficasProf. Albareda 1Granada18008Spain

## Abstract

Infections due to multidrug‐resistant bacteria represent a major global health challenge. To combat this problem, new antibiotics are urgently needed and some plant‐associated bacteria are a promising source. The rhizobacterium *Serratia plymuthica* A153 produces several bioactive secondary metabolites, including the anti‐oomycete and antifungal haterumalide, oocydin A and the broad spectrum polyamine antibiotic, zeamine. In this study, we show that A153 produces a second broad spectrum antibiotic, andrimid. Using genome sequencing, comparative genomics and mutagenesis, we defined new genes involved in andrimid (adm) biosynthesis. Both the expression of the *adm* gene cluster and regulation of andrimid synthesis were investigated. The biosynthetic cluster is operonic and its expression is modulated by various environmental cues, including temperature and carbon source. Analysis of the genome context of the *adm* operon revealed a gene encoding a predicted LysR‐type regulator, AdmX, apparently unique to *Serratia* strains. Mutagenesis and gene expression assays demonstrated that AdmX is a transcriptional activator of the *adm* gene cluster. At the post‐transcriptional level, the expression of the *adm* cluster is positively regulated by the RNA chaperone, Hfq, in an RpoS‐independent manner. Our results highlight the complexity of andrimid biosynthesis – an antibiotic with potential clinical and agricultural utility.

## Introduction

The discovery of antibiotics is one of the main milestones in the history of medicine. However, excessive overuse of antibiotics has encouraged the emergence of multidrug‐resistant bacteria, leading to a global increase in the spectrum of untreatable infections, which are currently responsible for around 50,000 annual deaths in Europe and the United States (Woodford *et al*., 2011; Blair *et al*., 2015). There is therefore an urgent need to identify new antibiotics, but efforts focussed on discovery and development of new antibiotics have met with only limited success (Lewis, 2013; Pidot *et al*., 2014). New platforms for antibiotic discovery include the generation of synthetic antimicrobials and development of species‐specific antibiotics (Fischbach and Walsh, 2009; Lewis, 2013; Liu *et al*., 2013; Pidot *et al*., 2014). It has been estimated that 90% of microbial natural products, and more than 99% of the total number of secondary metabolites, remain to be discovered. Consequently, recent approaches to antibiotic discovery include screening of microbes from new ecological niches and attempts at exploitation of previously uncultured microbes (Fischbach and Walsh, 2009).

Natural products (and their synthetic derivatives) comprise most of the antibiotics used clinically (Newman and Cragg, 2007), many of which are based on non‐ribosomal peptides and/or polyketides. Both families of secondary metabolites are synthesised by multifunctional enzymes, known as non‐ribosomal peptide synthetases (NRPSs) and polyketide synthases (PKSs), through sequential rounds of condensation of amino acids and acyl‐CoA building units, respectively (Sattely *et al*., 2008; Hertweck, 2009). The great structural diversity of non‐ribosomal peptides and polyketides results from the number of condensed building units and a range of pre‐ and post‐assembly processing reactions (Sattely *et al*., 2008; Hertweck, 2009). This chemical diversity is consequently reflected in a broad spectrum of biological activities (Sattely *et al*., 2008; Fischbach and Walsh, 2009; Hertweck, 2009; Pidot *et al*., 2014; Mousa and Raizada, 2015).

Some bacteria can devote up to 10% of their genomes to secondary metabolism (Udwary *et al*., 2007; Nett *et al*., 2009; Chowdhury *et al*., 2015). The biological synthesis of such metabolites can be energetically costly and so production is generally highly regulated (Coulthurst *et al*., 2005; Williamson *et al*., 2006; Liu *et al*., 2013). Biosynthetic gene clusters are often linked to their own regulatory genes (Chen *et al*., 2006; Zhao *et al*., 2010; Gurney and Thomas, 2011; Liu *et al*., 2013), the products of which are involved in sensing factors such as physiological state, population density and diverse environmental cues. As a result, the synthesis of the cognate secondary metabolite can be modulated appropriately. Quorum sensing regulatory circuits (Coulthurst *et al*., 2005; Williamson *et al*., 2006; Müller *et al*., 2009; Matilla *et al*., 2015), two‐component systems (Sola‐Landa *et al*., 2003; Haas and Défago, 2005; Williamson *et al*., 2006), orphan transcriptional regulators (Williamson *et al*., 2006; Lu *et al*., 2011; Klaponski *et al*., 2014) and post‐transcriptional regulators (Vogel and Luisi, 2011; Romeo *et al*., 2013) can all be involved in the regulatory complexity of bacterial secondary metabolite control.

The rhizosphere is one of the most complex environments on earth, with many organisms interacting and competing for nutrients and space (Lugtenberg and Kamilova, 2009; Mendes *et al*., 2013). Many rhizosphere microbes have evolved the capacity to synthesize bioactive secondary metabolites that allow them to efficiently antagonize diverse niche competitors (Berg *et al*., 2002; De Vleesschauwer and Höfte, 2007; Raaijmakers *et al*., 2009; Pidot *et al*., 2014; Mousa and Raizada, 2015). Consequently, this defines the rhizosphere as a habitat with great potential for exploitation as a source of new natural products with pharmacological, chemotherapeutic and agricultural applications.

*Serratia plymuthica* strains are near‐ubiquitous in nature but have been commonly isolated from soil and the rhizosphere of many economically important crops (De Vleesschauwer and Höfte, 2007). *Serratia plymuthica* strains possess great potential as biocontrol agents by antagonizing the growth of plant‐pathogens through the production of diverse bioactive secondary metabolites, siderophores and lytic enzymes (Alström, 2001; De Vleesschauwer and Höfte, 2007; Matilla *et al*., 2015). The strain used in this study, *Serratia plymuthica* A153, was isolated from the rhizosphere of wheat (Åstrom and Gerhardson, 1988) and it has been shown to possess bioactivity against fungi, oomycetes, bacteria and nematodes (Thaning *et al*., 2001; Matilla *et al*., 2012; Hellberg *et al*., 2015). These activities are mainly due to the synthesis of NRPS‐ and PKS‐based secondary metabolites, such as the haterumalide, oocydin A (Thaning *et al*., 2001), and the polyamine antibiotic, zeamine (Hellberg *et al*., 2015).

Our previous work showed that the strain A153, in addition to zeamine, produces an unidentified second antibacterial compound (Hellberg *et al*., 2015). In this study, we employed genome sequencing, comparative genomics and mutagenesis approaches to identify the genes involved in the biosynthesis of the unknown secondary metabolite. The regulation of the production of the antibacterial compound was also investigated and the results showed that the expression of the biosynthetic genes is tightly regulated at transcriptional and post‐transcriptional levels. Different environmental cues controlling the transcription of the biosynthetic genes were also identified.

## Results

### *Serratia plymuthica* A153 produces the hybrid non‐ribosomal peptide‐polyketide antibiotic, andrimid

**Figure 1:**
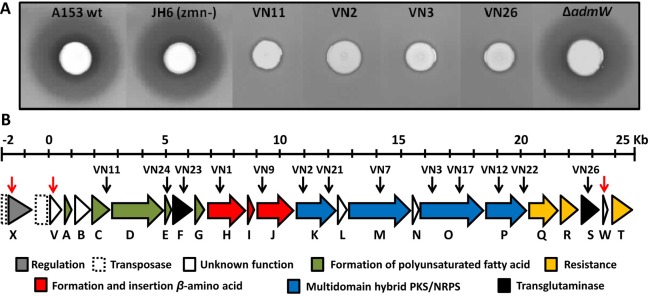
Identification and characterization of the andrimid gene cluster in *Serratia plymuthica* A153. A. Antibacterial activities against *Bacillus subtilis* of *Serratia plymuthica* A153, and derivative strains with mutations in the zeamine (*zmn*) and andrimid (*adm*) biosynthetic gene clusters. B. Genetic organization of the *adm* gene cluster in *S. plymuthica* A153. The same genetic organization was found in *S. marcescens* MSU97 and *S. marcescens* 90‐166 (Fig. S3). Location of the Tn‐KRCPN1 transposon insertions and in‐frame deletion mutants are indicated by black and red arrows, respectively. Colour code representing the functional category of each gene of the gene cluster is given where possible, based on the biosynthetic pathway for andrimid proposed by Jin *et al*. (2006). Genes *admV*, *admW* and *admX* were not previously associated with the regulation or biosynthesis of andrimid.

Characterization of the biocontrol rhizobacterium, *S. plymuthica* A153, showed that this strain possesses a strong bioactivity against *Bacillus subtilis* (Fig. 1A). The observed antibacterial activity was not associated with the production of other known bioactive secondary metabolites produced by A153, namely oocydin A (Matilla *et al*., 2012) or zeamine (Hellberg *et al*., 2015).

During the *in silico* analysis of the A153 genome sequence (Matilla *et al*., 2016) we identified at least five candidate biosynthetic PKS and NRPS gene clusters which could be responsible for the synthesis of the unknown antibacterial compound. To identify the genes responsible for this bioactivity, a random transposon insertion strain library was constructed and screened for mutants defective in antibacterial activity against *Bacillus subtilis*. Several transposon insertion mutants showing loss of antibacterial properties were isolated and all the insertions were transduced back into the wild type genetic background using the transducing phage ϕMAM1 (Matilla and Salmond, 2014) to confirm the link between transposon insertions and mutant phenotype. Random primed PCR confirmed that most of the transposons were located in a hybrid PKS/NRPS gene cluster described previously as responsible for the biosynthesis of the broad‐spectrum antibiotic, andrimid (Figs. 1 and Supporting information Fig. S1) (Jin *et al*., 2006).

We had access to another plant‐associated oocydin A producing strain, *Serratia marcescens* MSU97. This strain also showed strong antibacterial activity towards *B. subtilis* (Supporting information Fig. S2) and sequencing of its genome (Matilla and Salmond, unpubl. data) revealed that the andrimid (*adm*) gene cluster is also present in this plant epiphytic bacterium (Supporting information Fig. S3). We reported previously that MSU97 is recalcitrant to various genetic tools (Matilla *et al*., 2012) and attempts at isolating mutants defective in the *adm* gene cluster of this strain were unsuccessful.

### Comparative analyses of sequenced andrimid gene clusters

The biosynthesis of andrimid has been demonstrated in a broad range of bacteria (Fredenhagen *et al*., 1987; Long *et al*., 2005; Jin *et al*., 2006; Wietz *et al*., 2011; Sánchez *et al*., 2013). However, few *adm* gene clusters have been sequenced, including those of the marine bacteria, *Vibrionales* SWAT‐3 (PATRIC Genome ID 391574.12) and *Vibrio coralliilyticus* S2052 (Machado *et al*, 2015), and the plant‐associated enterobacterium *Pantoea agglomerans* Eh335 (Jin *et al*., 2006; GenBank^TM^ Accession No. AY192157.1). Additionally, our genomic analyses revealed that the *adm* gene cluster is also present in the recently sequenced rhizobacterium *Serratia marcescens* 90‐166 (Supporting information Figs. S3 and S4) (Jeong *et al*., 2015).

**Figure 2:**
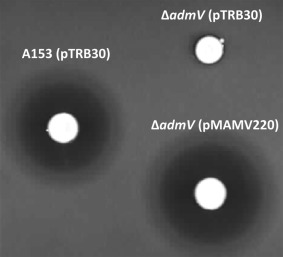
Role of the hypothetical protein AdmV in the biosynthesis of andrimid. Bioactivities against *Bacillus subtilis* of an in‐frame *admV* deletion mutant of *Serratia plymuthica* A153. Induction of the expression of the wild type proteins was done by addition of 1 mM of IPTG. The bioassays were repeated at least three times, and representative results are shown. Pictures were taken after 48 h of incubation at 25 °C.

Comparative analyses showed that the genomic context of the sequenced *adm* gene clusters in A153, MSU97, Eh335, S2052 and SWAT‐3 is completely different and, consequently, the upstream and downstream predicted ends of the biosynthetic clusters were assigned based only on their homologies (Supporting information Figs. S3 and S4). These analyses allowed the identification of a gene, designated *admV*, located immediately upstream of *admA*. *AdmV* was not previously associated with andrimid biosynthesis (Jin *et al*., 2006) and *in silico* analyses did not shed light on its putative function. However, we found that the gene *admV* is conserved in all *adm* gene clusters (Supporting information Figs. S3 and S4). To further investigate its role in the synthesis of andrimid, we constructed an in frame deletion mutant defective in *admV*, thereby avoiding polar effects in the expression of the downstream *adm* genes. The resulting mutant strain no longer exhibited antibacterial activity and the bioactivity could be complemented by the *in‐trans* expression of *AdmV* (Fig. 2).

The *adm* gene clusters of A153, MSU97, 90‐166, Eh335, SWAT‐3 and S2052 are 25.1, 24.9, 24.8, 24.7, 25.6 and 25.6 kb, respectively, and they are between 70.1% and 99.0% identical at the DNA level (Supporting information Fig. S4 and Table S1) suggesting that *adm* biosynthetic clusters may have been moved horizontally between the producing strains. In accordance with this hypothesis, the overall genomic G + C content of A153 (56.0%), MSU97 (58.9%), 90‐166 (59.1%), SWAT‐3 (44.5%) and S2052 (45.7%) is considerably different from the G + C content of their respective *adm* biosynthetic clusters, which are 45.8%, 47.6%, 46.27, 51.1% and 51.2%, respectively. Furthermore, remnant sequences of transposable genetic elements were found flanking the gene clusters of all the producing strains, although located in different regions in these loci (Supporting information Fig. S3).

In contrast, some remarkable differences were found between the six biosynthetic clusters. First, the intergenic regions of several *adm* contiguous genes are not conserved between the *adm* gene clusters (Supporting information Fig. S4) which could suggest differential regulation in the expression of the biosynthetic clusters. Second, the intergenic region between *admS* and *admT* is considerably larger in A153, MSU97 and 90‐166, and we identified a putative ORF, *admW*, in these three *Serratia* strains (Supporting information Figs. S3 and S4). However, the in frame deletion of *admW* did not alter the antibacterial activity of A153 (Fig. 1A). Finally, a gene encoding a LysR‐type transcriptional regulator was found upstream of *admT* in the *adm* gene clusters of *Vibrionales* SWAT‐3 and *Vibrio coralliilyticus* S2052 (Supporting information Fig. S3).

### The andrimid gene cluster consists of a large polycistronic unit

**Figure 3:**
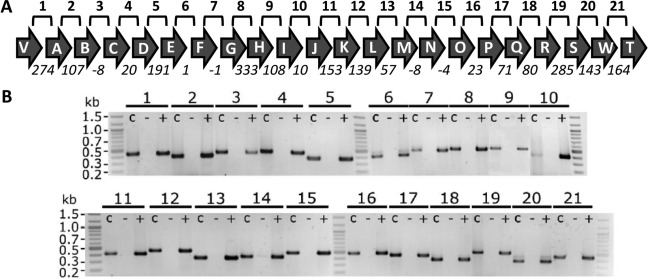
The andrimid gene cluster is organized as a polycistronic transcriptional unit. A. Schematic representation of the *adm* gene cluster in *Serratia plymuthica* A153. Lines labelled 1‐21 above the gene cluster represent the regions amplified by RT‐PCR and shown in B. Numbers below the arrows represent the intergenic distance in base pairs, and negative numbers indicate overlapping genes. B. Transcript analysis by RT‐PCR using primers designed to span the intergenic region between two adjacent genes. For each region, three PCR analyses were carried out: +, RT‐PCR on cDNA; −, negative control with no reverse transcriptase; c, positive control with genomic DNA as template. Culture samples for RNA isolation were taken at early stationary phase (Fig. 4).

The *adm* gene cluster in *Serratia* consists of 22 predicted ORFs and its genetic organization, together with the small intergenic distances between contiguous genes, suggests the presence of a single transcriptional unit (Figs. 3A and Supporting information Fig. S3). To further understand the biosynthesis of andrimid, we investigated its transcriptional regulation. With primers spanning the 3′ end of the upstream gene and the 5′ end of the contiguous downstream gene (Fig. 3A), we used reverse transcription‐PCR (RT‐PCR) to assess co‐transcription of the *adm* genes in *S. plymuthica* A153. PCR products were obtained across all intergenic regions covering the complete *adm* biosynthetic cluster, indicating the presence of one long polycistronic transcript (Fig. 3B) – although the possibility of internal promoters cannot be discarded.

### The transcription of the *adm* biosynthetic cluster is growth phase dependent

**Figure 4:**
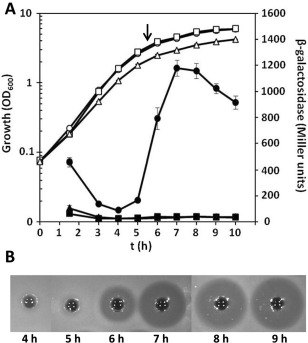
AdmX and Hfq regulate andrimid production by activating the expression of the *adm* biosynthetic gene cluster. A. β‐galactosidase activity (filled symbols) throughout growth measured from a chromosomal fusion *admK*::*lacZ* in *Serratia plymuthica* A153 LacZ (circles), and its Δ*admX* (squares) and Δ*hfq* (triangles) derivative strains in LB medium at 25 °C. Open symbols represent bacterial growth. Data are the mean and standard deviation of three biological replicates. Arrow, time point when samples for RT‐PCR and qPCR were taken (Figs. 3 and S6). B. Andrimid production by *S. plymuthica* A153 strain JH6 (zeamine negative) throughout growth in LB medium at 25 °C. For the assays, a *Bacillus subtilis* top agar lawn was prepared and 300 µl of filter‐sterilized supernatants were added to holes punched in *the Bacillus* bioassay plates.

**Figure 5:**
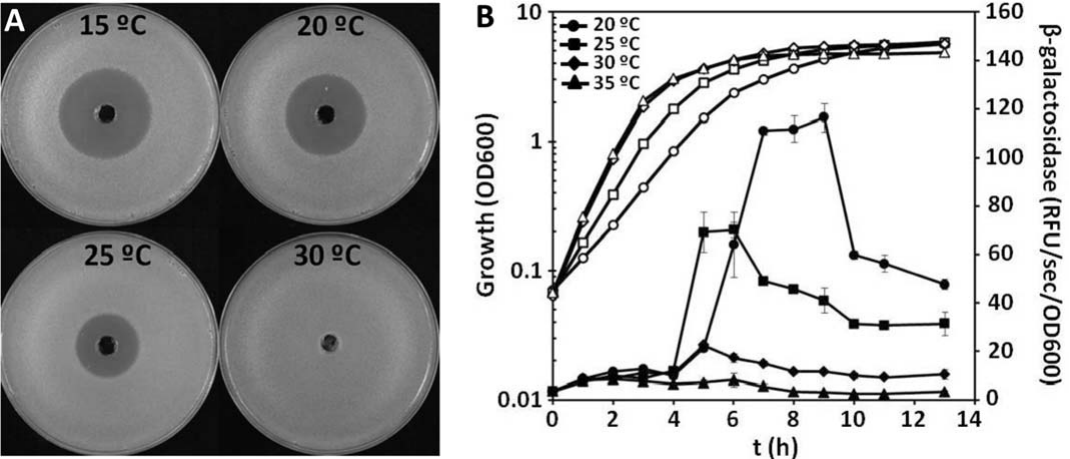
The production of andrimid in *Serratia plymuthica* A153 is temperature‐dependent and correlates with the expression of the *adm* gene cluster. A. Halos of antibiosis against *Bacillus subtilis* of filter‐sterilized supernatants of A153 strain JH6 (zeamine negative) grown in LB at different temperatures. The bioassays were repeated at least three times, and representative results are shown. B. β‐Galactosidase activity (filled symbols) throughout growth measured from the chromosomal fusion *admK*::*lacZ* in *Serratia plymuthica* A153 LacZ. Open symbols represent bacterial growth. Data are the mean and standard deviation of three biological replicates. Doubling times at 20, 25, 30 and 35 °C were 66.8 ± 0.4, 47.3 ± 0.7, 40.7 ± 0.4 and 40.5 ± 0.2 minutes respectively.

We identified several mutants in which the Tn‐KRCPN1 transposon generated β‐galactosidase transcriptional fusions (Table 1). Because our RT‐PCR analyses demonstrated the presence of an operonic *adm* biosynthetic cluster, we investigated transcription throughout growth in a Lac^‐^ derivative of A153. Using a chromosomal fusion located in the first multidomain PKS/NRPS‐encoding gene of the biosynthetic cluster (*admK*) our β‐galactosidase assays showed that the transcription of the *adm* operon started in late‐logarithmic phase of growth (Figs. 4A and 5B). Expression of the biosynthetic cluster correlated perfectly with the presence of andrimid in cell‐free supernatants (Fig. 4B).

**Table 1:** Bacteria and phages used in this study.

Bacteria/phage	Genotype or relevant characteristic^a^	Reference or source
*Escherichia coli* DH5α	*supE44 lacU169*(*Ø80lacZΔ M15*) *hsdR17* ( $r_{K}^{-}$ $m_{K}^{-}$) *recA1 endA1 gyrA96 thi‐1 relA1*	Woodcock *et al*. (1989)
*E. coli* CC118λpir	*araD*, Δ(*ara*, *leu*), Δ*lacZ*74, *pho*A20, *galK*, *thi‐1*, *rspE*, *rpoB*, *argE*, *recA1*, λ*pir*	Herrero *et al*. (1990)
*E. coli* HH26	Mobilizing strain for conjugal transfer	Kaniga *et al*. (1991)
*E. coli* β2163	F^‐^ RP4‐2‐Tc::Mu Δ*dapA*::(*erm‐pir*); Km^R^ Em^R^	Demarre *et al*. (2005)
*Serratia plymuthica* A153	Wild type, rhizosphere isolate	Hökeberg *et al*. (1997)
LacZ	A153 Δ*lacZ* (1470 bp Δ)	Matilla *et al*. (2015)
VN1	A153 transposon mutant *admH*::Tn‐KRCPN1*lacZ*; Km^R^	This study
VN2	A153 transposon mutant *admK*::Tn‐KRCPN1*lacZ*; Km^R^	This study
VN3	A153 transposon mutant *admO*::Tn‐KRCPN1*lacZ*; Km^R^	This study
VN7	A153 transposon mutant *admM*::Tn‐KRCPN1; Km^R^	This study
VN9	A153 transposon mutant *admJ*::Tn‐KRCPN1; Km^R^	This study
VN11	A153 transposon mutant *admC*::Tn‐KRCPN1*lacZ*; Km^R^	This study
VN12	A153 transposon mutant *admP*::Tn‐KRCPN1; Km^R^	This study
VN17	A153 transposon mutant *admO*::Tn‐KRCPN1; Km^R^	This study
VN21	A153 transposon mutant *admK*::Tn‐KRCPN1; Km^R^	This study
VN22	A153 transposon mutant *admP*::Tn‐KRCPN1*lacZ*; Km^R^	This study
VN23	A153 transposon mutant *admF*::Tn‐KRCPN1; Km^R^	This study
VN24	A153 transposon mutant *admE*::Tn‐KRCPN1*lacZ*; Km^R^	This study
VN26	A153 transposon mutant *admS*::Tn‐KRCPN1*lacZ*; Km^R^	This study
A153JH6	A153 Δ*lacZ*, *zmn13*::Tn‐KRCPN1; Zeamine^‐^; Km^R^	Hellberg *et al*. (2015)
ANDV	A153 Δ*admV* (336 bp Δ)	This study
ANDW	A153 Δ*admW* (150 bp Δ)	This study
ANDX	A153 Δ*admX* (789 bp Δ)	This study
XJH6	A153 Δ*admX, zmn13*::Tn‐KRCPN1; andrimid^‐^, zeamine^‐^; Km^R^	This study
ARpoS	A153 *rpoS*::Km; Km^R^	Matilla *et al*. (2015)
AHfq	A153 Δ*hfq*::Km (252 bp Δ); Km^R^	Matilla *et al*. (2015)
A153H	A153 Δ*hfq* (252 bp Δ)	This study
A153HL	A153 Δ*lacZ*, Δ*hfq*	This study
LVN2	A153 Δ*lacZ, admK*::Tn‐KRCPN1*lacZ*; generated by transduction using ϕMAM1; Km^R^	This study
HLVN2	A153 Δ*lacZ*, Δ*hfq, admK*::Tn‐KRCPN1*lacZ*; generated by transduction using ϕMAM1; Km^R^	This study
XLVN2	A153 Δ*lacZ*, Δ*admX, admK*::Tn‐KRCPN1*lacZ*; generated by transduction using ϕMAM1; Km^R^	This study
ASptI	A153 in‐frame *sptI* (483 bp Δ)	Matilla and Salmond (unpubl. data)
ASptR	A153 *sptR*::Km; Km^R^	Matilla and Salmond (unpubl. data)
ASplR	A153 *splR*::Km; Km^R^	Matilla and Salmond (unpubl. data)
ASpsR	A153 *spsR*::Km; Km^R^	Matilla and Salmond (unpubl. data)
A153C	A153 in‐frame *csrB* (267 bp Δ)	Matilla and Salmond (unpubl. data)
*Serratia marcescens* MSU97	Wild type, plant epiphyte, pigmented	Strobel *et al*. (1999)
*Bacillus subtilis* JH642	*pheA1 trpC2*	J.A. Hoch
*Dickeya solani* MK10	Wild type, plant pathogen	Pritchard *et al*. (2013)
*Xanthomonas campestris* pv. *campestris*	Wild type, plant pathogen	R. Penyalver
**Phages**		
ϕMAM1	Generalized transducing phage for *S. plymuthica* A153	Matilla and Salmond (2014)

**a.** The following abbreviations are used: Km, kanamycin; Tc, tetracycline; Em, erythromycin.

### Temperature and carbon source regulate the transcription of the *adm* operon

The biosynthesis of secondary metabolites can be energetically costly and other secondary metabolites produced by *Serratia* strains have been shown to be highly regulated by various environmental cues (Williamson *et al*., 2006; Coulthurst *et al*., 2005). To shed light on the regulation of andrimid production in A153, we investigated the impact of different environmental parameters in the transcription of the *adm* operon.

At the optimal growth temperature (30 °C) for A153, andrimid production was abolished (Fig. 5A). However, as the temperature decreased, a gradual increase in the production of andrimid was observed, with higher production levels at 15 °C than at 25 °C (Fig. 5A). To examine whether the increase in andrimid production was reflected in the expression of the *adm* gene cluster, we evaluated the impact of temperature on the transcription of the biosynthetic cluster. β‐galactosidase assays showed that the transcription was also thermoregulated (Fig. 5B). In accordance with this, no *adm* expression was observed at temperatures above 30 °C, confirming the tight correlation between andrimid production and transcription of the biosynthetic operon (Fig. 5B).

**Figure 6:**
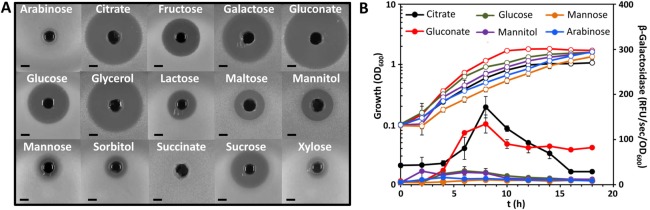
Effect of carbon source on the production of andrimid and expression of the *adm* gene cluster in *Serratia plymuthica* A153. A. Halos of antibiosis against *Bacillus subtilis* of filter‐sterilized supernatants of A153 strain JH6 (zeamine negative) grown in minimal medium with different carbon sources. The bioassays were repeated at least three times, and representative results are shown. All the carbon sources were used at a final concentration of 15 mM. With the exception of lactose, all carbon sources used are frequently found in plant root exudates. Bars, 5 mm. B. β‐galactosidase activity (filled symbols) throughout growth measured from the chromosomal fusion *admK*::*lacZ* in *Serratia plymuthica* A153 LacZ in minimal medium with different carbon sources. Open symbols represent bacterial growth. Data are the mean and standard deviation of three biological replicates. Growth and doubling times of A153 in all the carbon sources used are shown in Fig. S5.

*Serratia plymuthica* A153 was originally isolated from the rhizosphere (Astrom and Gerhardson, 1988), a soil environment which is under the direct influence of plant‐root exudates. The composition of root exudates is chemically complex but quantitatively it consists mainly of carbon‐based compounds, primarily organic acids and sugars (Uren, 2007; Badri and Vivanco, 2009; Suzuki *et al*., 2009). Thus, we investigated the production of andrimid in response to different carbon sources found in plant root exudates. Our results showed that andrimid is differentially produced depending on carbon source. Maximal antibacterial activity was observed in the presence of citrate, gluconate or glycerol whereas no activity was detected in the presence of arabinose or succinate as sole carbon sources (Fig. 6A). Although the growth rates of A153 varied between the tested carbon sources (Supporting information Fig. S5), the observed antibacterial activity was not correlated with the growth rate *per se* or the final optical density reached in each of the culture media. Consistent with these observations, higher transcriptional levels of the *adm* operon were observed in media containing citrate or gluconate, whereas no transcription was seen in arabinose media (Fig. 6B), confirming that both transcriptional activity and andrimid production, were carbon source‐dependent.

Finally, we also evaluated the production of andrimid at different pH, aeration conditions and NaCl concentrations, but no impacts on antibacterial synthesis were observed (not shown).

### The LysR‐type regulator, AdmX, activates andrimid production

**Figure 7:**
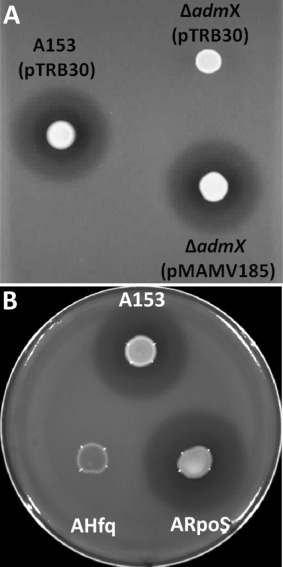
The LysR‐type regulator AdmX, and the RNA chaperone Hfq positively regulate the biosynthesis of andrimid. Bioactivities against *Bacillus subtillis* of *S. plymuthica* A153 and derivative strains are shown (A, B). In frame deletion of *admX* was functionally complemented by the *in trans* expression of AdmX using the pQE80L‐based vector, pMAMV185 (A). Induction of the expression of the AdmX was done by addition of 0.1 mM of IPTG. Complementation of the Δ*hfq* strain was done using a pQE‐80L‐based vector (Fig. S8). The bioassays were repeated at least three times, and representative results are shown. Pictures were taken after 48 h of incubation at 25 °C.

Analysis of the genomic context of the *adm* gene clusters in A153, MSU97, 90‐166, Eh335, S2052 and SWAT‐3 revealed a 2 kb region upstream of *admV* which was highly conserved in the *Serratia* strains (Supporting information Figs. S3 and S4). This region contained a gene encoding a putative LysR‐type transcriptional regulator (LTTR; Supporting information Figs. S3 and S4). The family of LTTRs comprises one of the largest classes of transcriptional regulators in bacteria, functioning either as repressors or activators of the transcription of their target genes (Maddocks and Oyston, 2008). To investigate the potential role of the identified LTTR in andrimid biosynthesis, we engineered an in frame deletion mutant in *S. plymuthica* A153. The resulting mutant did not inhibit growth of *B. subtilis* but antibacterial activity was fully restored by *in trans* expression of the LTTR encoding gene (Fig. 7A). These results suggested that the regulator, designated AdmX, is responsible for activating transcription of the *adm* gene cluster and β‐galactosidase assays confirmed that expression of the biosynthetic cluster was abolished in an *admX*‐deficient strain (Fig. 4A). These results were supported by qPCR analyses showing decreased *adm* transcript levels in the A153 Δ*admX* strain (Supporting information Fig. S6).

To assess the transcription of *admX* throughout growth, we constructed a transcriptional fusion of the *admX* promoter to the reporter gene *lacZ*. β‐galactosidase assays showed that the transcription of *admX* started in late‐logarithmic phase of growth, correlating with transcription of the *adm* biosynthetic cluster (Supporting information Fig. S7). We explored the effect of AdmX on expression of its cognate gene. However, the transcription of *admX* remained unaltered in an *admX* mutant strain (Supporting information Fig. S7). Finally, we also investigated whether andrimid thermoregulation is mediated by AdmX. Previous studies showed that the expression of another LTTR (PecT), and consequently the expression of its target genes, is thermoregulated (Hérault *et al*., 2014). However, no differences in transcripts levels of *admX* were observed (using qPCR analysis) at 25, 30 and 37 °C (data not shown).

### The chaperone Hfq regulates the expression of the *adm* gene cluster

The biosynthesis of secondary metabolites is frequently regulated at post‐transcriptional level and, in gammaproteobacteria, this is mainly mediated by the Csr/Rsm (Romeo *et al*., 2013) and Hfq (Vogel and Luisi, 2011) systems. To assess a potential role of these post‐transcriptional regulatory systems, we analysed the production of andrimid in different genetic backgrounds in *S. plymuthica* A153. A mutant with a deletion in the non‐coding sRNA, *csrB*, showed the same antibacterial activity as the wild type strain (not shown). However, in frame deletion of *hfq* led to complete loss of andrimid production (Fig. 7B) and this phenotype was partially complemented by the *in trans* expression of *hfq* (Supporting information Fig. S8).

β‐galactosidase assays were performed to assess the expression of the *adm* gene cluster throughout growth in A153 Δ*hfq*. The results showed that transcription of the andrimid operon was abolished in a *hfq*‐deficient strain, indicating that the chaperone Hfq positively regulates expression of the *adm* biosynthetic cluster (Fig. 4A). qPCR analyses confirmed that the transcriptional levels of the *adm* gene cluster were reduced by 99.9% in an *hfq*‐deficient background (Supporting information Fig. S6). To investigate whether Hfq regulates *admX* expression, qPCR experiments were also performed. However, *admX* transcript levels were unaltered in the Δ*hfq* mutant (Supporting information Fig. S6).

The expression of the *adm* gene cluster starts in late‐logarithmic phase of growth (Fig. 4) and so we investigated whether quorum sensing (QS) could be regulating andrimid production. A153 carries *sptI, sptR*, *splR* and *spsR* genes encoding LuxI‐ and LuxR‐type proteins very similar to those of the three QS systems identified in the taxonomically related rhizobacterium, *S. plymuthica* G3 (Liu *et al*., 2011; Duan *et al*., 2012). However mutations in each of these four genes in A153 had no observable impacts on antibacterial activity (not shown). Similarly, the role of the stationary phase sigma factor, RpoS, in the production of andrimid was also investigated, but the antibacterial activity of an *rpoS* mutant was indistinguishable from that of the wild type strain (Fig. 7B).

## Discussion

Since the golden age of antimicrobial discovery in the 1940–1960s, there has been a diminution in the rate of discovery of new antibiotics and this problem has been exacerbated by the progressive emergence worldwide of antibiotic‐resistant bacteria (Lewis, 2013; Blair *et al*., 2015). However, there are many antibiotics described in the literature that, although not currently exploited, could prove to be lead molecules for drug discovery programmes leading to clinical or agricultural utility. The hybrid NRPS/PKS antibacterial compound, andrimid, was first isolated in the late 1980s from a brown planthopper intracellular symbiont belonging to the *Enterobacter* genus (Fredenhagen *et al*., 1987). After its discovery, three related antibacterial compounds, moiramides A‐C (Supporting information Fig. S1), were also isolated from *Pseudomonas fluorescens* (Needham *et al*., 1994).

Andrimid is a nanomolar inhibitor of the bacterial acetyl‐CoA carboxylase (Freiberg *et al*., 2004), an enzyme responsible for the conversion of acetyl‐CoA to malonyl‐CoA in the first committed step for the synthesis of fatty acids (Harwood, 2007). The discovery of the andrimid target, together with its unusual chemistry, has stimulated interest in the biochemistry and genetics of the antibiotic. The andrimid gene cluster was first described almost a decade ago and a model for its biosynthesis proposed (Jin *et al*., 2006). In this model, the function for most of the Adm proteins was predicted and, in some cases, later demonstrated biochemically (Fortin *et al*., 2007; Magarvey *et al*., 2008; Ratnayake *et al*., 2011). However, the roles of the hypothetical proteins AdmB, AdmL and AdmN in the synthesis of andrimid remain unknown. In this study, we redefined the length of the *adm* gene cluster by identifying a new hypothetical protein encoding gene, *admV*, located immediately upstream of *admA* and forming part of the *adm* operon (Figs. 3 and Supporting information Fig. S3). Using mutagenesis and complementation analyses we demonstrated that *AdmV* is required for the biosynthesis of andrimid. Our bioinformatic analyses did not categorically clarify the function of *AdmV*. However, *in silico* studies predicted three alpha helices with a high content of positively charged amino acids at the C‐terminal end, suggesting that *AdmV* may be a DNA binding protein.

Andrimid has been shown to be a broad‐spectrum antibacterial compound that acts on Gram‐positive and Gram‐negative bacteria. It was first isolated after it was shown to have potent activity against the different pathovars of the phytopathogen, *Xanthomonas campestris* (Fredenhagen *et al*., 1987) but further research showed that it is also active against bacterial pathogens belonging to *Bacillus*, *Enterococcus, Escherichia, Salmonella*, *Staphylococcus, Vibrio* and *Yersinia* genera (Needham *et al*., 1994; Singh *et al*. 1997; Long *et al*., 2005; Wietz *et al*., 2010; Sánchez *et al*., 2013). Resistance to andrimid is dependent on multidrug efflux pumps (Freiberg *et al*., 2004; Jin *et al*., 2006) or the presence of andrimid‐resistant acetyl‐CoA carboxyltransferases (ACC) hypothesized to show decreased affinity for the inhibitor (Liu *et al*., 2008). Interestingly, we showed that the plant‐pathogen *Agrobacterium tumefaciens* and multiple rhizobacteria strains in the family Enterobacteriaceae (i.e., *Kluyvera*, *Pantoea*, *Serratia*, *Weeksella*, *Xenorhabdus*, and the emerging phytopathogen, *Dickeya solani*) (Supporting information Figs. S9 and S10) are sensitive to andrimid. However, the differential sensitivities to the antibiotic of these plant‐associated strains could also indicate the presence of weaker intrinsic resistance mechanism(s) of different efficiencies.

NRPS and PKS constitute the main enzymatic source of secondary metabolites in bacteria; 36% of which are generated by hybrid NRPS/PKS gene clusters (Wang *et al*., 2014). Secondary metabolites have been associated with important roles in the physiology and development of the bacterial host, but are also implicated in overcoming competitors in the same nutritional niche (Liu *et al*., 2013; Pidot *et al*., 2014; Wang *et al*., 2014; Mousa and Raizada, 2015). The synthesis *in situ* of andrimid has been demonstrated in a sponge‐associated bacterium (Oclarit *et al*., 1994; Long *et al*., 2005) and its production is favoured over that of other metabolites in conditions mimicking natural environments ‐ suggesting an important ecophysiological role of andrimid (Wietz *et al*., 2011). Consistent with this view, even sub‐lethal concentrations of andrimid can promote a negative chemotactic response (in sensitive competitors) away from the antibiotic, thereby enhancing competitiveness of the producers (Graff *et al*., 2013). In *S. plymuthica* A153, the synthesis of andrimid is carbon source‐dependent and induced in the presence of sugars and organic acids commonly found in plant root exudates (Fig. 6). Carbon source has been shown to regulate antibiotic synthesis at both molecular and physiological levels and, in general, rapidly metabolized carbon sources are involved in repression of bacterial secondary metabolites (Sánchez *et al*., 2010). However, our results did not show a correlation between preferred carbon sources and andrimid production (Figs. 6 and S5). Importantly, in A153, the production of the antibiotic zeamine is high in the presence of carbon sources which show low andrimid production (i.e., arabinose, sorbitol and succinate) whereas the synthesis of zeamine is repressed by carbon sources that stimulate andrimid production (i.e., citrate, gluconic acid and glycerol) (Fig. 6; Hellberg *et al*., 2015). This “mirror image” metabolic regulation of andrimid and zeamine production when presented with different environmental carbon sources could be beneficial in maintaining the capacity of A153 to overcome bacterial competitors in its natural niche, the rhizosphere, where specific carbon source availability is likely to fluctuate.

Soil bacteria are subjected to daily and seasonal abiotic variations and temperature is considered a key abiotic factor influencing bacterial metabolic activity – which may therefore modulate inter‐ and intra‐specific interactions between microbes. The results reported here show that andrimid synthesis in A153 is thermoregulated, with enhanced antibiotic production at lower temperature. Previous studies also showed that andrimid production in *Vibrio* (Long *et al*., 2005; Wietz *et al*., 2011) and *Serratia* strains (Sánchez *et al*., 2013) is also thermosensitive. In this study, we demonstrate that the observed thermoregulation is exerted at the transcriptional level (Fig. 5) and in a AdmX‐independent fashion. During the analysis of the bioactive properties of A153, we showed that the production of the haterumalide, oocydin A, is also thermosensitive (Matilla and Salmond, unpubl. data) and this may indicate that a common control pathway is modulating the thermoregulation of the biosynthesis of secondary metabolites in A153. The ecological role of this thermosensitivity is unknown, although it could be related to competitiveness and efficient colonization of specific niches by the producing rhizobacterium, *S. plymuthica* A153.

To date, most of the published research on andrimid has focussed on its biochemistry, but the regulation of its biosynthesis has attracted little attention. The first *adm* gene cluster was identified in *Pantoea agglomerans* but no regulatory proteins were found in this biosynthetic cluster (Jin *et al*., 2006). However, our analysis of the genomic context of the andrimid gene cluster in A153, MSU97 and 90‐166, revealed a LysR‐type regulator‐encoding gene, *admX*, which was highly conserved in these producing strains (Supporting information Figs. S3 and S4). Mutagenesis, complementation and gene expression analyses demonstrated that AdmX is a positive regulator of andrimid biosynthesis (Figs. 4 and 7A). AdmX is a 305 amino acid protein composed of a helix‐turn‐helix motif‐containing DNA‐binding domain and a probable effector binding domain at the N‐terminal and C‐terminal regions, respectively (Supporting information Fig. S11). By analogy with other LTTR structures (Ezezika *et al*., 2007; Monferrer *et al*., 2010; Devesse *et al*., 2011), AdmX is proposed to possess a potential effector binding site located in between the two lobes of the effector binding domain (Supporting information Fig. S11). Inspection of a homology model shows that this site is primarily composed of amino acids with hydrophobic side chains (Supporting information Fig. S11) and further investigations are necessary to identify candidate effector molecules.

In general, LysR transcriptional regulators activate the expression of their target genes while negatively autoregulating their own transcription (Maddocks and Oyston, 2008). LTTRs have been shown to regulate genes involved in metabolism, virulence, motility, chemotaxis, quorum sensing and biofilm formation (Maddocks and Oyston, 2008). However, only a limited number of LTTRs have been found to regulate the biosynthesis of secondary metabolites – such as actinorhodin (Mao *et al*., 2013), phenazines (Klaponski *et al*., 2014), pyoluteorin (Li *et al*., 2012), ralfuranones (Kai *et al*., 2014) and undecylprodigiosin (Mao *et al*., 2013). LTTRs are broadly distributed within the prokaryotic kingdom, suggesting dissemination and acquisition by horizontal gene transfer (HGT; Maddocks and Oyston, 2008). In accordance with this notion, sequences reminiscent of transposases were found flanking *admX* (Supporting information Figs. S3 and S4) and the G + C content of the *admX* genes in A153, MSU97 and 90‐166 is considerably higher than that of their respective *adm* gene clusters. Although AdmX seems to be restricted to the *adm* biosynthetic clusters present in *Serratia* strains, BLAST analyses showed that AdmX is up to 84% identical (90% similar) to an orphan LysR‐type regulator highly conserved within *Enterobacter* and *Klebsiella* genera, again suggesting that this LTTR‐encoding gene may have been acquired by HGT. Interestingly, we found an LTTR‐encoding gene immediately upstream of *admT* in the *adm* gene clusters of *Vibrio coralliilyticus* S2052 and *Vibrionales* SWAT‐3 (Supporting information Fig. S3), perhaps indicating an important regulatory role in the expression of the *adm* gene clusters in these strains. It has been proposed that the fragmented andrimid biosynthetic pathway reflects a recent evolutionary origin (Magarvey *et al*., 2008) and so the insertion of the LTTR‐encoding genes in the *adm* gene clusters of *Vibrio* and *Serratia* may represent a step forward in the evolution of these biosynthetic clusters.

The post‐transcriptional regulation of the *adm* gene cluster in A153 was also investigated and our results showed that the RNA binding protein, Hfq, positively regulates the expression of the andrimid operon. Previous studies of *Serratia* strains showed that the synthesis of secondary metabolites such as a carbapenem antibiotic (Wilf *et al*., 2011), prodigiosin (Wilf *et al*., 2011) and pyrrolnitrin (Zhou *et al*., 2012) is regulated by Hfq, and we recently showed that Hfq positively regulates the expression of the oocydin A (Matilla *et al*., 2015) and zeamine (Hellberg *et al*., 2015) gene clusters in *S. plymuthica* A153. It is known that Hfq also stimulates *rpoS* translation (Vogel and Luisi, 2011) and the deletion of *hfq* results in reduced *rpoS* transcripts levels in A153 (Matilla *et al*., 2015). However, contrary to our observations on regulation of oocydin A in A153 (Matilla *et al*., 2015), Hfq‐mediated regulation of andrimid is independent of RpoS (Fig. 7B), as observed in other *Serratia* strains for the biosynthesis of zeamine (Hellberg *et al*., 2015), carbapenem (Wilf and Salmond, 2012) and prodigiosin (Wilf and Salmond, 2012) antibiotics.

In summary, we have shown that the plant‐associated bacterium, *Serratia plymuthica* A153, produces the broad spectrum antibacterial compound, andrimid. Comparative genomics, molecular genetics and transcriptional approaches led us to re‐define the borders of the *adm* gene clusters and identify new genes involved in the biosynthesis and regulation of andrimid. Further, *in vivo* assays expanded the spectrum of bacterial strains which are sensitive to andrimid. For the first time, the regulation of the biosynthesis of andrimid was investigated at transcriptional and post‐transcriptional levels. Future research will provide information about the effectors recognized by AdmX and such knowledge, coupled with the work described in this study, may encourage the use of plant‐associated andrimid‐producing strains as biocontrol agents in sustainable agriculture strategies.

## Experimental procedures

### Bacterial strains, culture media and growth conditions

Bacterial strains used in this study are listed in Table 1 and Supporting Information Table S2. *Serratia, Bacillus, Agrobacterium, Dickeya, Kluyvera, Pantoea, Weeksella, Xanthomonas, Xenorhabdus, Yersinia* and their derivative strains were grown routinely at 30 °C, unless otherwise indicated, in Luria Broth (LB; 5 g yeast extract l^−1^,10 g Bacto tryptone l^−1^ and 5 g NaCl l^−1^) or minimal medium (0.1%, w/v, (NH_4_)_2_SO_4_, 0.41 mM MgSO_4_, 40 mM K_2_HPO_4_, 14.7 mM KH_2_PO_4_, pH 6.9–7.1) with glucose (0.2%; w/v) as carbon source, unless otherwise indicated. *Escherichia coli* strains were grown at 37 °C in LB. *Escherichia coli* DH5α was used as a host for gene cloning. Media for propagation of *E. coli* β2163 were supplemented with 300 µM 2,6‐diaminopimelic acid. When appropriate, antibiotics were used at the following final concentrations (in µg ml^−1^): ampicillin, 100; chloramphenicol, 25; kanamycin, 25 (*E. coli* strains) and 50 (*Serratia* strains); streptomycin, 50; tetracycline, 10. Sucrose was added to a final concentration of 10% (w/v) when required to select derivatives that had undergone a second crossover event during marker‐exchange mutagenesis. Bacterial growth (OD_600 nm_) was measured on a Unicam Heλios spectrophotometer at 600 nm, 1 cm path length.

### *In vitro* nucleic acid techniques and bioinformatic analyses

Plasmid DNA was isolated using the Anachem Keyprep plasmid kit. For DNA digestion, the manufacturer's instructions were followed (New England Biolabs, Roche and Fermentas). Separated DNA fragments were recovered from agarose gels using the Anachem gel recovery kit. Ligation reactions and total DNA extraction were performed as previously described (Sambrook *et al*., 1989). Competent cells were prepared using calcium chloride and transformations were performed by standard protocols (Sambrook *et al*., 1989). Phusion® high fidelity DNA polymerase (New England Biolabs) was used in the amplification of PCR fragments for cloning. PCR reactions were purified using the Anachem PCR Clean‐up kit. PCR fragments were verified by DNA sequencing that was carried out at the University of Cambridge DNA Sequencing Facility (Cambridge, UK) or at the Institute of Parasitology and Biomedicine Lopez*‐*Neyra (CSIC; Granada, Spain). Sequence comparison analyses were performed employing the wgVISTA online tool (Frazer *et al*., 2004). Open reading frames (ORFs) in the andrimid gene clusters were predicted using Glimmer 3.0 (Delcher *et al*., 1999). Blast analyses were used for the functional gene assignment. Protein domain organization was identified using the NCBI conserved domains database. Multiple sequence alignments were carried out with ClustalW2 (European Bioinformatics Institute). Artemis software (Wellcome Trust Sanger Institute) was used to visualize genomic sequences.

### Random transposon mutagenesis

Random transposon mutagenesis of *S. plymuthica* A153 using Tn‐KRCPN1 was performed by biparental conjugation mating using *Escherichia coli* β2163, as described previously (Matilla *et al*., 2012). In total, three thousand kanamycin‐resistant insertion mutants were screened for their antibacterial activity against *Bacillus subtilis* using dual drop culture bioassays. Auxotrophic mutants were discarded and insertion mutations were transduced into the wild type strain A153 using phage ϕMAM1 (Matilla and Salmond, 2014). The insertion site of transposon Tn‐KRCPN1 in mutants of interest was determined using random primed PCR following the method described previously (Matilla *et al*., 2012) and using primers described in Supporting Information Table S3.

### Antibacterial assays

Antibiotic activity was tested using agar lawn assays. Briefly, indicator plates for andrimid production contained a 0.8% LB agar (LBA) top lawn containing 200 µl of an overnight culture of the bacterial strain to test. Five microliters of overnight cultures of the andrimid‐producing strains were spotted on the surface of the indicator agar lawn and incubated for 48 h at 25 °C, unless otherwise indicated. To determine andrimid levels in bacterial supernatants, culture samples were taken, bacterial cells were pelleted by centrifugation (10,000×*g*, 10 min), and the supernatant was filtered (0.2 µm). Three hundred microliters of the filter‐sterilized supernatant were added to wells cut into the LBA plate and incubated at 25 °C for 24 h. All experiments were repeated at least three times.

### Construction of strains and plasmids

Chromosomal mutants of *Serratia plymuthica* strains were constructed by homologous recombination using derivative plasmids of the suicide vector pKNG101. These plasmids, which are listed in Supporting Information Table S4, were confirmed by DNA sequencing and they carried mutant in‐frame deletions for the replacement of wild type genes in the chromosome. Primers used in this study are listed in Supporting Information Table S3. In all cases, plasmids for mutagenesis were transferred to *S. plymuthica* strains by triparental conjugation using *E. coli* CC118λ*pir* and *E. coli* HH26 (pNJ500) as helper. The plasmids for the construction of the in‐frame deletion mutants were generated by amplifying the up‐ and downstream flanking regions of the gene, or domain to be deleted. The resulting PCR products were digested with the enzymes specified in Supporting Information Table S4 and ligated in a three‐way ligation into pUC18Not, previously cloned into the marker exchange vector pKNG101. The in‐frame deletion mutant strains ANDX, ANDV and ANDW were generated using plasmids pMAMV175, pMAMV191 and pMAMV192, respectively. Mutant strains defective in *hfq*, A153H and A153HL, were constructed using plasmid pMAMV193. All relevant mutations were confirmed by PCR and sequencing.

For the construction of the complementing plasmids, the genes were amplified using primers described in Supporting Information Table S3 and cloned into pTRB30. All the inserts were confirmed by PCR and sequencing. Complementing plasmids were used to transform A153 by electroporation.

### Genetic complementation assays

Complementation of mutations was carried out by the introduction of a wild type copy of the corresponding mutated gene *in trans* on plasmid pTRB30. For the complementation assays, LBA containing the appropriate antibiotic (to maintain the plasmid) and isopropyl‐β‐D‐thiogalactopyranoside (IPTG) at 0.1 or 1 mM were added to holes punched in *Bacillus subtilis* bioassay plates. Then, 5 μl of overnight cultures of the selected strains were spotted on the surface of the LBA containing the antibiotic and IPTG and were incubated at 25 °C for 2 days.

### Generalized transduction

The generalized transducing viunalikevirus, ϕMAM1, was used for transduction of chromosomal mutations, as described previously (Matilla and Salmond, 2014).

### β‐Galactosidase assays

Expression of the *lacZ* reporter gene was performed using the fluorogenic substrate 4‐methylumbelliferyl β‐D‐galactoside (Melford Cat No. M1095) at a final concentration of 0.125 mg ml^−1^, as described previously (Ramsay, 2013). Samples were measured in a SpectraMax Gemini XPS fluorescence microplate reader (Molecular Devices) using the following settings: excitation 360 nm, emission 450 nm, cut‐off 435 nm, reading every 30 s for 20 min at 37 °C. β‐Galactosidase activity was expressed as relative fluorescent units s^−1^ and normalize to the OD_600 nm_ of the corresponding sample. Alternatively, β‐galactosidase activity was measured as described previously (Miller, 1972) using 2‐Nitrophenyl β‐D‐galactopyranoside (ONPG; Sigma–Aldrich Cat No. N1127) as substrate. All the transcriptional fusion assays were carried out using *S. plymuthica* A153 LacZ (control) or derived mutants.

### RNA extraction, cDNA synthesis, reverse transcription‐PCR (RT‐PCR) and quantitative real time PCR analyses

RNA was extracted from early stationary phase cultures grown in LB medium using an RNeasy mini kit (Qiagen) according to the manufacturer's instructions. RNA concentration was determined spectrophotometrically and RNA integrity was assessed by agarose gel electrophoresis. Genomic DNA contamination was eliminated by treating total RNA with Turbo DNA‐free (Ambion). The synthesis of cDNA was performed using random hexamers (GE Healthcare) and SuperScript II reverse transcriptase (Invitrogen) in a 30 µl reaction with 2 µg of total RNA and incubation at 42 °C for 2 h. As negative control the reaction was performed omitting the reverse transcriptase. For the RT‐PCR analysis, the equivalent of 50 ng of total RNA was subjected to PCR amplification using primers to amplify across the junctions (Supporting Information Table S3). Positive and negative control PCR reactions were performed using genomic DNA and no‐RT cDNA samples, respectively, as templates. PCR conditions consisted of 30 cycles of denaturation for 1 min at 94 °C, annealing for 1 min at 62 °C, and extension for 40 s at 72 °C. qPCRs were performed as described previously (Burr *et al*., 2006) using primers specific for *admX* and *admV* (Supporting Information Table S3). qPCR amplifications were performed using an MyiQ^TM^2 Two‐Color Real‐Time PCR Detection System (Bio‐Rad). To confirm the absence of contaminating genomic DNA, control PCRs were carried out using no RT cDNA samples as templates. Melting curve analyses were conducted to ensure amplification of a single product. The relative gene expression was calculated using the critical threshold (ΔΔCt) method (Pfaffl, 2001) and using 16S rRNA as the internal control to normalize the data.

## Supporting information

Additional Supporting Information may be found in the online version of this article at the publisher's web‐site:

**Fig. S1.** Structures of andrimid and moiramide B. The structure of andrimid consists of an unsaturated fatty acid chain, a pyrrolidinedione ring, a valine and glycine derived β‐ketoamide and the amino acid β‐phenylalanine. Chemical synthesis studies showed that the fatty acid chain and β‐phenylalanine are involved in bacterial cell penetration of the antibiotic, whereas the pyrrolidinedione head and the β‐ ketoamide moiety are responsible for the antibacterial activity (Pohlmann *et al*., 2005; Freiberg *et al*., 2006).
**Fig. S2.** Antibacterial activity of *Serratia marcescens* MSU97 against *Bacillus subtilis*.
**Fig. S3.** Schematic representation of the andrimid gene clusters of *Serratia marcescens* MSU97, *Serratia marcescens* 90‐166, *Pantoea agglomerans* Eh335, *Vibrio coralliilyticus* S2052 and *Vibrionales* bacterium SWAT‐3. Numbers below the arrows represent the intergenic distance between contiguous genes, with negative numbers indicate overlapping genes
**Fig. S4.** DNA homology between the andrimid gene cluster of *Serratia plymuthica* A153 and the andrimid gene clusters of the other producing strains. A, Schematic representation of the *adm* gene cluster in *Serratia* strains. B‐F, Alignments representing the percentage of DNA homology between the *adm* gene cluster of A153 and those of *S. marcescens* MSU97 (B), *S. marcescens* 90‐166 (C), *Pantoea agglomerans* Eh335 (D) *Vibrio coralliilyticus* S2052 (E) and *Vibrionales* SWAT‐3 (F). Alignments were performed using wgVISTA (Frazer *et al*., 2004).
**Fig. S5.** Growth of *Serratia plymuthica* A153 in minimal medium with different carbon sources. Growth curves showing the doubling time in sorbitol (181.2 ± 1 min), mannitol (153 ± 1 min), fructose (145.8 ± 2 min), galactose (115.2 ± 1 min), mannose (181.6 ± 2 min), lactose (413.4 ± 6 min), xylose (208.2 ± 2 min), succinic acid (142.8 ± 1 min), maltose (145.2 ± 1 min), sucrose (106.2 ± 1 min), glucose (115.2 ± 1 min), glycerol (121 ± 1 min), gluconic acid (96.6 ± 1 min), arabinose (235.2 ± 2 min) and citrate (158.7 ± 3 min) as sole carbon source. Data are the mean and standard deviation of three biological replicates. The assays were done at 25 ºC with shaking at 200 rpm.
**Fig S6.** Impact of Hfq (A) and AdmX (B) on the expression of *admV* and *admX*. Quantitative real‐time PCR was used to measure transcript levels of *admV* (grey bars) and *admX* (white bars) in *Serratia plymuthica* A153, and derivative strains. The values showed the average expression relative to wild type expression. The arrow in Fig. 4A indicates the time point when the samples for qPCR were taken. The data are the mean and standard deviation of three biological replicates.
**Fig. S7.** AdmX transcription correlates with the expression of the andrimid gene cluster. Transcription of the *admX* (P*admX*::*lacZ*; pMAMV244) promoter fusion throughout growth in *Serratia plymuthica* A153 strains. β‐Galactosidase activity (filled symbols) and growth curves (open symbols) were determined in LacZ (red) and Δ*admX* (blue) in LB medium at 25 °C. A153 wt harbouring the empty reporter plasmid (black) was used as negative control in the assays. Data are the mean and standard deviation of three biological replicates.
**Fig. S8.** Genetic complementation of *Serratia plymuthica* A153 strain A153H. Expression of *hfq in trans* in A153 Δ*hfq* restored andrimid production and therefore the antibacterial activity against *Bacillus subtilis*. Induction of Hfq expression was done by addition of 0.1 mM of IPTG. The bioassays were repeated at least three times, and a representative figure is shown. Pictures were taken after 48 h of incubation at 25 °C.
**Fig. S9.** Sensitivities of different bacterial strains to the antibiotic andrimid. Bioactivities of *Serratia plymuthica* A153 and the non‐andrimid producing mutant of A153, VN2, against ecologically different bacterial strains. For the assays, an indicator top agar lawn was prepared as described in “Experimental procedures,” and 5 μl overnight cultures of the A153 strains were spotted on the surface of the bacterial indicator agar lawns. The bioassays were repeated three times, and representative results are shown. Pictures were taken after 48 h of incubation at 25 °C. The strains used are described in Table 1 and supplementary Table S2.
**Fig. S10.** Andrimid shows antibacterial activity against *Bacillus subtilis*, *Dickeya solani* and *Xanthomonas campestris* pv. *campestris*. Recovery of viable *Bacillus*, *Dickeya* and *Xanthomonas* cells grown in the presence of A153 JH6 (andrimid positive, zeamine negative) and A153 XJH6 (andrimid and zeamine negative) supernatants. The values showed the percentage of viable cells in the presence of JH6 supernatants relative to the number of viable cells in the presence of XJH6 supernatants. For the assays, overnight bacterial cultures of *Bacillus*, *Dickeya* and *Xanthomonas* were adjusted to an optical density at 600 nm (OD600) of 0.1 and grown at 30 ºC with orbital shaking (225 rpm). At an OD600 of 0.4, 10 mL of the bacterial culture was removed and pelleted by centrifugation at 4,000 x *g* for 10 min at room temperature. The pellet was resuspended in 5 ml of 2X LB and 5 ml supernatants of an overnight culture of A153 JH6 or A153 XJH6 were added to the bacterial culture. Samples were taken after 5 and 10 h of incubation and the number of colony forming units (CFU) were determined. Data are the mean and standard deviation of three biological replicates.
**Fig. S11.** Homology model of AdmX. The model was generated by the Geno3D modeling algorithm (Combet *et al*., 2000) and the structure of the BenM transcriptional regulator (PDB ID 3K1N) as template. The site for the binding of potential effector molecules is indicated.
**Table S1.** Identity at DNA level of the andrimid gene clusters between producing strains.
**Table S2.** Additional bacterial strains used in this study.
**Table S3.** Oligonucleotides used in this study.
**Table S4.** Plasmids used in this study.
