# Supplementary material for: Biosynthesis of the acetyl‐CoA carboxylase‐inhibiting antibiotic, andrimid in Serratia is regulated by Hfq and the LysR‐type transcriptional regulator, AdmX
Source: Environ Microbiol. 2016 May 25;18(11):3635–50. doi: 10.1111/1462-2920.13241 (PMC5216899; doi:10.1111/1462-2920.13241)
Supplement: Supplementary file 1 — Fig. S1. Structures of andrimid and moiramide B. The structure of andrimid consists of an unsaturated fatty acid chain, a pyrrolidinedione ring, a valine and glycine derived β‐ketoamide and the amino acid β‐phenylalanine. Chemical synthesis studies showed that the fatty acid chain and β‐phenylalanine are involved in bacterial cell penetration of the antibiotic, whereas the pyrrolidinedione head and the β‐ ketoamide moiety are responsible for the antibacterial activity (Pohlmann et al., 2005; Freiberg et al., 2006). Fig. S2. Antibacterial activity of Serratia marcescens MSU97 against Bacillus subtilis. Fig. S3. Schematic representation of the andrimid gene clusters of Serratia marcescens MSU97, Serratia marcescens 90‐166, Pantoea agglomerans Eh335, Vibrio coralliilyticus S2052 and Vibrionales bacterium SWAT‐3. Numbers below the arrows represent the intergenic distance between contiguous genes, with negative numbers indicate overlapping genes Fig. S4. DNA homology between the andrimid gene cluster of Serratia plymuthica A153 and the andrimid gene clusters of the other producing strains. A, Schematic representation of the adm gene cluster in Serratia strains. B‐F, Alignments representing the percentage of DNA homology between the adm gene cluster of A153 and those of S. marcescens MSU97 (B), S. marcescens 90‐166 (C), Pantoea agglomerans Eh335 (D) Vibrio coralliilyticus S2052 (E) and Vibrionales SWAT‐3 (F). Alignments were performed using wgVISTA (Frazer et al., 2004). Fig. S5. Growth of Serratia plymuthica A153 in minimal medium with different carbon sources. Growth curves showing the doubling time in sorbitol (181.2 ± 1 min), mannitol (153 ± 1 min), fructose (145.8 ± 2 min), galactose (115.2 ± 1 min), mannose (181.6 ± 2 min), lactose (413.4 ± 6 min), xylose (208.2 ± 2 min), succinic acid (142.8 ± 1 min), maltose (145.2 ± 1 min), sucrose (106.2 ± 1 min), glucose (115.2 ± 1 min), glycerol (121 ± 1 min), gluconic acid (96.6 ± 1 min), arabinose (235.2 ± 2 min) and citrate [file EMI-18-3635-s001.pdf]

Supporting information

Biosynthesis of the acetyl-CoA carboxylase-inhibiting antibiotic, andrimid, in *Serratia* is regulated by Hfq and the LysR-type transcriptional regulator, AdmX.

Miguel A. Matilla[ab]*, Veronika Nogellova[a], Bertrand Morel[b], Tino Krell[b] and George P.C. Salmond[a]*

[a]Department of Biochemistry, University of Cambridge, Tennis Court Road, Cambridge, UK, CB2 1QW.

[b]Department of Environmental Protection, Estación Experimental del Zaidín, Consejo Superior de Investigaciones Científicas, Prof. Albareda 1, Granada, Spain, 18008

Running title: *Regulation of the synthesis of andrimid*

*Address correspondence to George P.C. Salmond, Department of Biochemistry, University of Cambridge, Tennis Court Road, Cambridge, UK, CB2 1QW. Tel: +44 (0)1223 333650; E-mail: gpcs2@cam.ac.uk

*Address correspondence to Miguel A. Matilla, Department of Environmental Protection, Estación Experimental del Zaidín, Consejo Superior de Investigaciones Científicas, Prof. Albareda 1, Granada, Spain, 18008. Tel: +34 958 181600; Fax: + 34 958 135740; E-mail: miguel.matilla@eez.csic.es

Supplementary Table S1: Identity at DNA level of the andrimid gene clusters between producing strains.

Strain	DNA Homology (%)					
	**Eh335**	**MSU97**	**A153**	**SWAT-3**	**90-166**	**S2052**
***Pantoea agglomerans* Eh335**	**X**	83.2	84.2	70.1	81.7	70.1
***Serratia marcescens* MSU97**	83.2	**X**	86.4	71.8	89.1	71.2
***Serratia plymuthica* A153**	84.2	86.4	**X**	71.4	84.8	71.4
***Vibrio* SWAT-3**	70.1	71.8	71.4	**X**	70.7	99.0
***Serratia marcescens* 90-166**	81.7	89.1	84.8	70.7	**X**	70.7
***Vibrio coralliilyticus* S2052**	70.1	71.2	71.4	99.0	70.7	**X**

Supplementary Table S2. Additional bacterial strains used in this study.

**Bacteria**	**Genotype or relevant characteristic**[a]	**Reference or source**
Agrobacterium tumefaciens C58	Wild type; Plant pathogen	Wood *et al*., 2001
Bacillus thuringiensis subsp. *kurstaki* strain HD73	Wild type; Toxic to lepidopteran larvae	Liu *et al*., (2013)
Dickeya solani MK10	Wild type, plant pathogen	Pritchard *et al*., (2013)
Dickeya solani MK16	Wild type, plant pathogen	Pritchard *et al*., (2013)
Dickeya solani IPO 2222	Wild type, plant pathogen	Pritchard *et al*., (2013)
Escherichia coli EPI100-T1R	*F⁻ mcrA* Δ*(mrr-hsdRMS-mcrBC) Φ80dlacZΔM15* Δ*lacX74 recA1 endA1 araD139* Δ*(ara, leu)7697 galU galK λ⁻ rpsL* (Str^R) *nupG trfA tonA*	Epicentre
Escherichia coli OP50	Uracil auxotroph	Brenner (1974)
Kluyvera cryorescens 2Kr27	Wild type; Rhizosphere isolate	Berg *et al*., (2002)
Pantoea agglomerans 10Bp14	Wild type; Rhizosphere isolate	Berg *et al*., (2002)
Pantoea agglomerans 9Rz4	Wild type; Rhizosphere isolate	Berg *et al*., (2002)
Serratia marcescens 12	*Serratia marcescens* strain 3888; wild type, clinical isolate	Aucken and Pitt (1998)
Serratia proteomaculans 3Rc15	Wild type; Rhizosphere isolate	Berg *et al*., (2002)
Serratia proteomaculans 9Bp4	Wild type; Rhizosphere isolate	Berg *et al*., (2002)
Serratia plymuthica 4Rx5	Wild type; Rhizosphere isolate	Berg *et al*., (2002)
Weeksella zoohelcum 5Rr4	Wild type; Rhizosphere isolate	Berg *et al*., (2002)
Weeksella zoohelcum 8Rx9	Wild type; Rhizosphere isolate	Berg *et al*., (2002)
Xenorhabdus luminescens 3Rp5	Wild type; Rhizosphere isolate	Berg *et al*., (2002)
Xanthomonas campestris pv. *campestris*	Wild type; Plant pathogen	R. Penyalver
Yersinia enterocolitica	Wild type	Lab stock

Supplementary Table S3. Oligonucleotides used in this study.

Number	Name	Sequence (5´- 3')	Description	Source
1	PF106	GACCACACGTCGACTAGTGCNNNNNNNNNNAGAG	Random primed PCR primer 1	Fineran *et al*., (2005)
2	PF107	GACCACACGTCGACTAGTGCNNNNNNNNNNACGCC	Random primed PCR primer 2	Fineran *et al*., (2005)
3	PF108	GACCACACGTCGACTAGTGCNNNNNNNNNNGATAC	Random primed PCR primer 3	Fineran *et al*., (2005)
4	PF109	GACCACACGTCGACTAGTGC	Random primed PCR adapter primer	Fineran *et al*., (2005)
5	MAMV1-KRCPN1	GGAATTGATCCGGTGGATG	TnKRCPN1 specific primer	Matilla *et al*., (2012)
6	MAMV2-KRCPN1	GCATAAAGCTTGCTCAATCAATCAC	TnKRCPN1 specific primer	Matilla *et al*., (2012)
7	admVA-F	AATTCATGTCCCCACTCGC	RT-PCR mapping forward primer product 1	This study
8	admVA-R	AATTCATGTCCCCACTCGC	RT-PCR mapping reverse primer product 1	This study
9	admAB-F	AATACAGCATTGACCTGGGG	RT-PCR mapping forward primer product 2	This study
10	admAB-R	TTATCGTATGTCCGGCCATC	RT-PCR mapping reverse primer product 2	This study
11	admBC-F	GGATCTCAATAGGCAGGGC	RT-PCR mapping forward primer product 3	This study
12	admBC-R	CGGCATTGTTCACCAGAAC	RT-PCR mapping reverse primer product 3	This study
13	admCD-F	CCGGGCTACATCGATACTGA	RT-PCR mapping forward primer product 4	This study
14	admCD-R	CGTGGCTCTTAAGGCAAAAA	RT-PCR mapping reverse primer product 4	This study
15	admDE-F	CGATGGGCATTAAAAGCAAC	RT-PCR mapping forward primer product 5	This study
16	admDE-R	CAGCAGTACACCCGGAACA	RT-PCR mapping reverse primer product 5	This study
17	admEF-F	GTCGAATATTGCCGGGATT	RT-PCR mapping forward primer product 6	This study
18	admEF-R	TCTTAAATCAAGCCCGCGT	RT-PCR mapping reverse primer product 6	This study
19	admFG-F	GGTGCGGTGATTTAGACCTC	RT-PCR mapping forward primer product 7	This study
20	admFG-R	TGAATTCCTGCTGCCTGAG	RT-PCR mapping reverse primer product 7	This study
21	admGH-F	AAGCGTTTACCAGCACCAAC	RT-PCR mapping forward primer product 8	This study
22	admGH-R	CTCGTCATTTCGGCTTGAG	RT-PCR mapping reverse primer product 8	This study
23	admHI-F	GGACATTGTCTCTTTTGGGC	RT-PCR mapping forward primer product 9	This study
24	admHI-R	TCCACCGAATCGAGGATATC	RT-PCR mapping reverse primer product 9	This study
25	admIJ-F	GATTACGGCCTGTCATTTGG	RT-PCR mapping forward primer product 10	This study
26	admIJ-R	CTGACATGATCATCGCCAA	RT-PCR mapping reverse primer product 10	This study
27	admJK-F	TGATCCCAAATATCTTCGCC	RT-PCR mapping forward primer product 11	This study

28	admJK-R	CCTGATAAGAATACGCGGCA	RT-PCR mapping reverse primer product 11	This study
29	admKL-F	CGATTCTTCTGGACTGTCGG	RT-PCR mapping forward primer product 12	This study
30	admKL-R	TGTTAACGACTGGGCAGTTG	RT-PCR mapping reverse primer product 12	This study
31	admLM-F	GTCAGCCCCGTGATATTGAA	RT-PCR mapping forward primer product 13	This study
32	admLM-R	CAAACTGATCGACTCCCTCC	RT-PCR mapping reverse primer product 13	This study
33	admMN-F	GATAATCCCCGGCTCAATCT	RT-PCR mapping forward primer product 14	This study
34	admMN-R	TCGCATTGTCACCATCATTC	RT-PCR mapping reverse primer product 14	This study
35	admNO-F	TCGAAACAAGACAGAGACGC	RT-PCR mapping forward primer product 15	This study
36	admNO-R	GGTAGGTATGATTGCTGCCC	RT-PCR mapping reverse primer product 15	This study
37	admOP-F	TCTCTGGTAGGGAGAAACGG	RT-PCR mapping forward primer product 16	This study
38	admOP-R	ATAATACAACGCCCTCGACG	RT-PCR mapping reverse primer product 16	This study
39	admPQ-F	CTGTGGGTAAGATCTTGGGC	RT-PCR mapping forward primer product 17	This study
40	admPQ-R	GCGGCAGCTAAGGTGTAACT	RT-PCR mapping reverse primer product 17	This study
41	admQR-F	TGTTTAGTGGTTTGGCTGCC	RT-PCR mapping forward primer product 18	This study
42	admQR-R	ATTCCACACCAAAATCCGAG	RT-PCR mapping reverse primer product 18	This study
43	admRS-F	GGATATGTGACAAATCGGCA	RT-PCR mapping forward primer product 19	This study
44	admRS-R	CGTCATGCTTTGTTGGTTTG	RT-PCR mapping reverse primer product 19	This study
45	admSW-F	TGAGATTGCGGATGAAATGA	RT-PCR mapping forward primer product 20	This study
46	admSW-R	ATACGAACAGATAGCCCGCA	RT-PCR mapping reverse primer product 20	This study
47	admWT-F	GCTGATTGTCGCGATTATTG	RT-PCR mapping forward primer product 21	This study
48	admWT-R	ATGTGGTGATCGCACTTCG	RT-PCR mapping reverse primer product 21	This study
49	admV-KpnI-F	TAATGGTACCTAGTATCGTGGGTTGTAGTCC	Forward primer to clone upstream flanking region of *admV* for in-frame deletion	This study
50	admV-BamHI-R	TAATGGATCCCGTAGAACCATACTATAGCACTCC	Reverse primer to clone upstream flanking region of *admV* for in-frame deletion	This study
51	admV-BamHI-F	TAATGGATCCTAACTGGACCCAGTTCGTG	Forward primer to clone downstream flanking region of *admV* for in-frame deletion	This study
52	admV-HindIII-R	TAATAAGCTTTCATGTTATCCAGCGCTATC	Reverse primer to clone downstream flanking region of *admV* for in-frame deletion	This study
53	admW-EcoRI-F	TAATGAATTCATGGCACTCGACATGATGAG	Forward primer to clone upstream flanking region of *admW* for in-frame deletion	This study
54	admW-BamHI-R	TAATGGATCCTTATAGACGGCAAAGTGGCG	Reverse primer to clone upstream flanking region of *admW* for in-frame deletion	This study
55	admW-BamHI-F	TAATGGATCCGCCACGAGCGTAGTAAAGC	Forward primer to clone downstream flanking region of *admW* for in-frame deletion	This study

56	admW-HindIII-R	TAATAAGCTTGTCAGCACAGAGATGTACGG	Reverse primer to clone downstream flanking region of *admW* for in-frame deletion	This study
57	admX-EcoRI-F	TAATGAATTCGGTCGTATCGGCTGACA	Forward primer to clone upstream flanking region of *admX* for in-frame deletion	This study
58	admX-BamHI-R	TAATGGATCCGCCGCATGTCATCACGGTG	Reverse primer to clone upstream flanking region of *admX* for in-frame deletion	This study
59	admX-BamHI-F	TAATGGATCCCGGCTACGTAACGAGCTCAG	Forward primer to clone downstream flanking region of *admX* for in-frame deletion	This study
60	admX-HindIII-R	TAATAAGCTTCCTGGCGGAATTCATGG	Reverse primer to clone downstream flanking region of *admX* for in-frame deletion	This study
61	AdmX-RBS-EcoRI-F	TAATGAATTCACCTAGGATGAACAGTCTATGAAAC	Forward primer to clone *admX* into pTRB30.	This study
62	AdmX-EcoRI-R	TAATGAATTCCGCTTATATGAAAGCATTTAGACTGG	Reverse primer to clone *admX* into pTRB30	This study
63	AdmV-BamHI-F	TAATGGATCCGGCACCGTCAGGTGCCCTTTTG	Forward primer to clone *admV* into pTRB30.	This study
64	AdmV-KpnI-R	TAATGGTACCTCGATCGCAAAACGGCTGAAATGAG	Reverse primer to clone *admV* into pTRB30	This study
65	AdmX-qPCR-F	GGGTGCCGTCGAGATTGATAG	Forward primer for qRT-PCR	This study
66	AdmX-qPCR-R	ATGTCGGTGATGCAGCATCC	Reverse primer for qRT-PCR	This study
67	AdmV-qPCR-F	TTGGCTCCGAAGGCAACAAG	Forward primer for qRT-PCR	This study
68	AdmV-qPCR-R	ACATCACGCAGATCCGTACC	Reverse primer for qRT-PCR	This study
69	16SA153-qPCR-F	ACTGAGACACGGTCCAGACT	Forward primer for qRT-PCR	This study
70	16SA153-qPCR-R	TTAGCCGGTGCTTCTTCTGC	Reverse primer for qRT-PCR	This study
71	PadmX-KpnI-F	TAATGGTACCATGCCACCTACATACTGC	Forward primer to clone promoter region of *admX* into pMP220	This study
72	PadmX-PstI-R	TAATCTGCAGCATAGACTGTTCATCCTAGGTTATG	Reverse primer to clone promoter region of *admX* into pMP220	This study

Supplementary Table S4. Plasmids used in this study.

Plasmid	Relevant characteristic[a]	Source
pKNG101	Sm^R; *oriR6K mob sacBR*	Kaniga *et al*., (1991)
pUC18Not	Ap^R; identical to pUC18 but with two NotI sites flanking pUC18 polylinker	Herrero *et al*., (1990)
pNJ5000	Tc^R; Mobilizing plasmid used in marker exchange	Grinter (1983)
pKCPRN1	Km^R, Tc^R; Derivative of pDS1028*uidA* with the *uidA* and *cat* genes replaced with *lacZ* and *km* genes	K. Roberts PhD
pTRB30	Km^R; pQE-80L (Qiagen) based expression vector, Ap^R resistance cassette replaced by Km^R. IPTG-inducible promoter, ColE1 origin.	T. Blower
pMP220	Tc^R; *oriRK2 'lacZ*	Spaink *et al*., (1987)
pMAMV117	Ap^R; 1.4-kb PCR product containing a 252 bp deletion of *hfq* of A153 inserted into the EcoRI/SphI sites of pUC18Not	Matilla *et al*., (2015)
pMAMV193	Sm^R; 1.5-kb NotI fragment of pMAMV117 was cloned at the same site in pKNG101	This study
pMAMV144	Ap^R; 1.4-kb PCR product containing a 789 bp in frame deletion of *admX* of A153 inserted into the EcoRI/HindIII sites of pUC18Not	This study
pMAMV175	Sm^R; 1.5-kb NotI fragment of pMAMV144 was cloned at the same site in pKNG101	This study
pMAMV189	Ap^R; 1.5-kb PCR product containing a 336 bp in frame deletion of *admV* of A153 inserted into the KpnI/HindIII sites of pUC18Not	This study
pMAMV191	Sm^R; 1.6-kb NotI fragment of pMAMV189 was cloned at the same site in pKNG101	This study
pMAMV190	Ap^R; 1.5-kb PCR product containing a 150 bp in frame deletion of *admW* of A153 inserted into the EcoRI/HindIII sites of pUC18Not	This study
pMAMV192	Sm^R; 1.6-kb NotI fragment of pMAMV190 was cloned at the same site in pKNG101	This study
pMAMV185	Km^R; *admX* gene was cloned into the EcoRI site of pTRB30	This study
pMAMV220	Km^R; *admV* gene was cloned into the EcoRI site of pTRB30	This study
pJEEUH13	Km^R; *hfq* gene cloned into the expression vector pTRB30	Hellberg *et al*., (2015)
pMAMV244	Tc^R; *admX* promoter region was cloned into the KpnI/PstI sites of pMP220	This study

[a]Ap, ampicillin; Km, kanamycin; Sm, streptomycin; Tc, tetracycline.

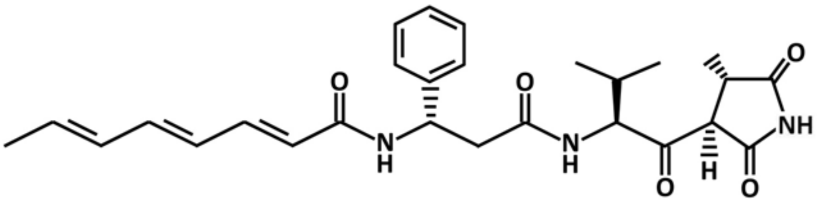

Andrimid

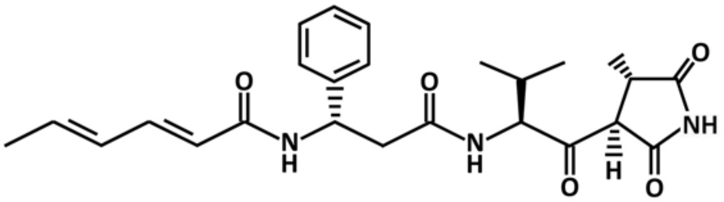

Moiramide B

Supplementary Fig. S1: Structures of andrimid and moiramide B. The structure of andrimid consists of an unsaturated fatty acid chain, a pyrrolidinedione ring, a valine and glycine derived β-ketoamide and the amino acid β-phenylalanine. Chemical synthesis studies showed that the fatty acid chain and β-phenylalanine are involved in bacterial cell penetration of the antibiotic, whereas the pyrrolidinedione head and the β-ketoamide moiety are responsible for the antibacterial activity (Pohlmann *et al.,* 2005; Freiberg *et al*., 2006).

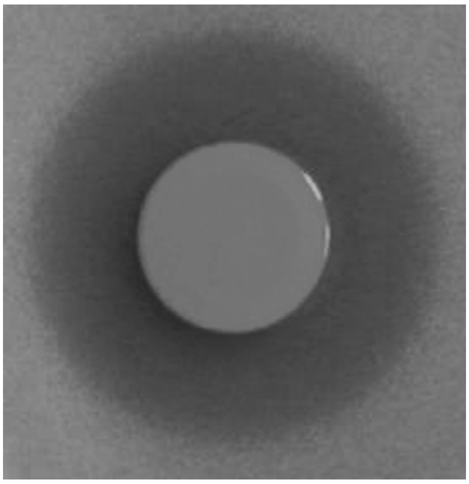

Supplementary Fig. S2. Antibacterial activity of *Serratia marcescens* MSU97 against *Bacillus subtilis*.

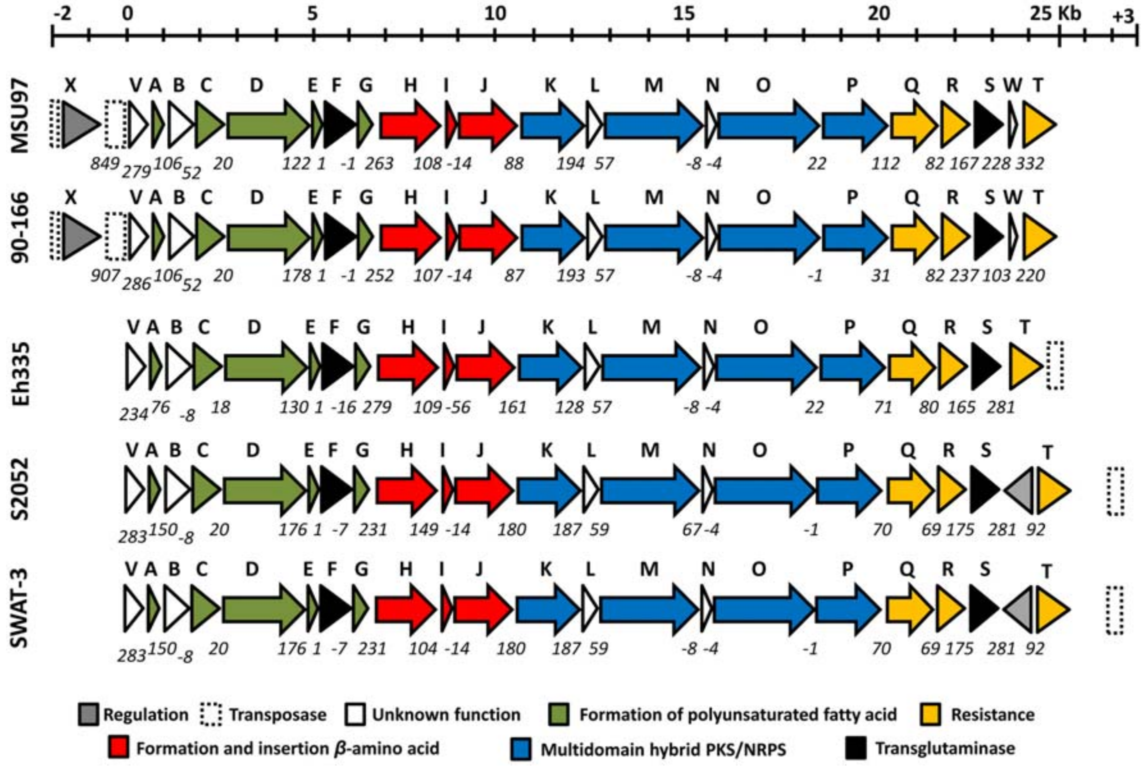

Supplementary Fig. S3. Schematic representation of the andrimid gene clusters of *Serratia marcescens* MSU97, *Serratia marcescens* 90-166, *Pantoea agglomerans* Eh335, *Vibrio coralliilyticus* S2052 and *Vibrionales* bacterium SWAT-3. Numbers below the arrows represent the intergenic distance between contiguous genes, with negative numbers indicate overlapping genes.

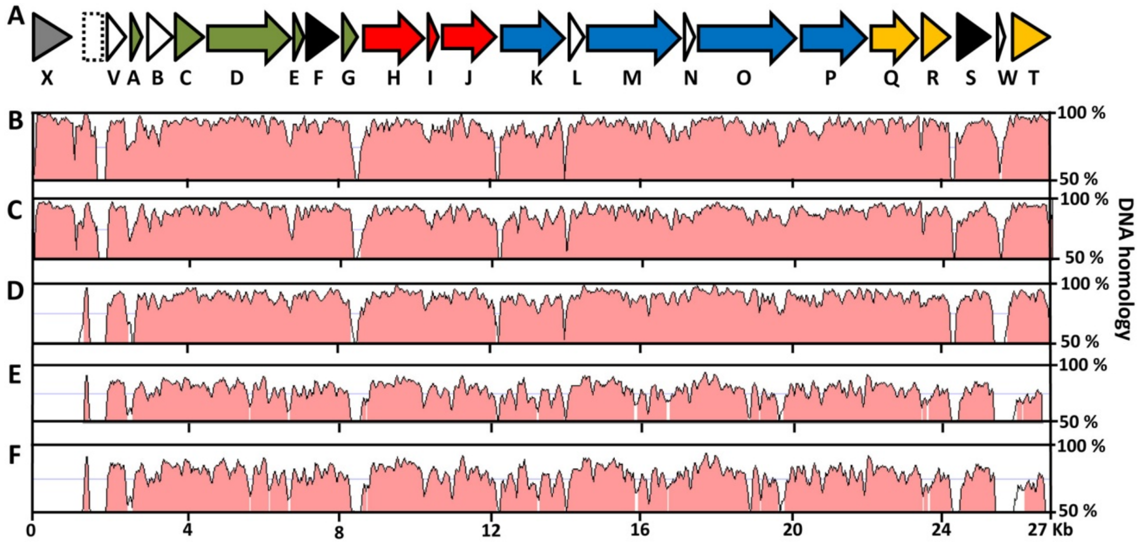

Fig. S4. DNA homology between the andrimid gene cluster of *Serratia plymuthica* A153 and the andrimid gene clusters of the other producing strains. A, Schematic representation of the *adm* gene cluster in *Serratia* strains. **B-F**, Alignments representing the percentage of DNA homology between the *adm* gene cluster of A153 and those of *S. marcescens* MSU97 (B), *S. marcescens* 90-166 (C), *Pantoea agglomerans* Eh335 (D) *Vibrio coralliilyticus* S2052 (E) and *Vibrionales* SWAT-3 (F). Alignments were performed using wgVISTA (Frazer *et al*., 2004).

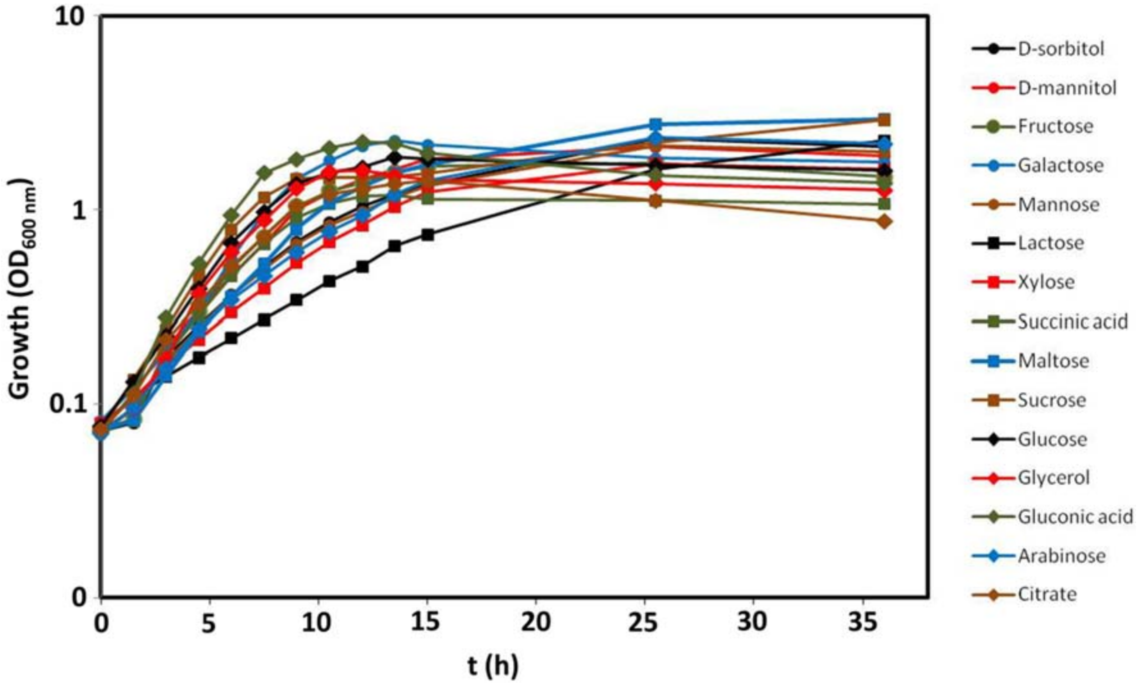

Supplementary Fig. S5: Growth of *Serratia plymuthica* A153 in minimal medium with different carbon sources. Growth curves showing the doubling time in sorbitol (181.2 ± 1 min), mannitol (153 ± 1 min), fructose (145.8 ± 2 min), galactose (115.2 ± 1 min), mannose (181.6 ± 2 min), lactose (413.4 ± 6 min), xylose (208.2 ± 2 min), succinic acid (142.8 ± 1 min), maltose (145.2 ± 1 min), sucrose (106.2 ± 1 min), glucose (115.2 ± 1 min), glycerol (121 ± 1 min), gluconic acid (96.6 ± 1 min), arabinose (235.2 ± 2 min) and citrate (158. 7 ± 3 min) as sole carbon source. Data are the mean and standard deviation of three biological replicates. The assays were done at 25 ºC with shaking at 200 rpm.

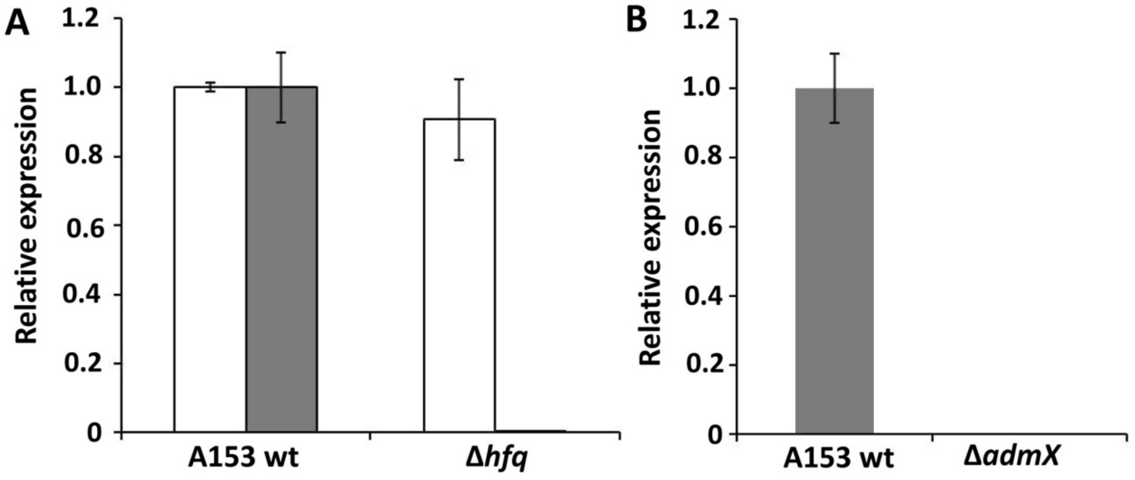

Supplementary Fig S6: Impact of Hfq (A) and AdmX (B) on the expression of *admV* and *admX*. Quantitative real-time PCR was used to measure transcript levels of *admV* (grey bars) and *admX* (white bars) in *Serratia plymuthica* A153, and derivative strains. The values showed the average expression relative to wild type expression. The arrow in Fig. 4A indicates the time point when the samples for qPCR were taken. The data are the mean and standard deviation of three biological replicates.

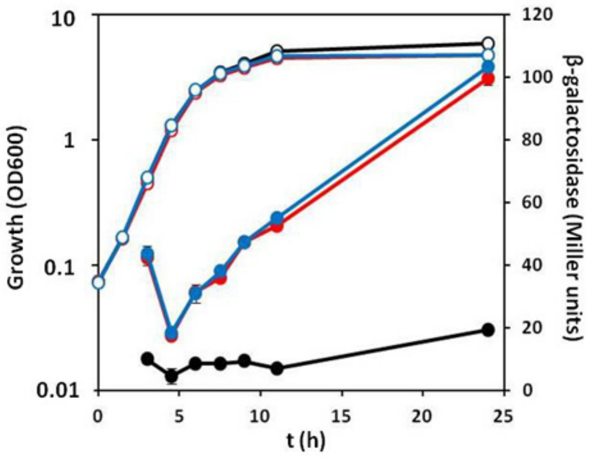

Supplementary Fig. S7. AdmX transcription correlates with the expression of the andrimid gene cluster. Transcription of the *admX* (P_{admX}::*lacZ*; pMAMV244) promoter fusion throughout growth in *Serratia plymuthica* A153 strains. β-Galactosidase activity (filled symbols) and growth curves (open symbols) were determined in LacZ (red) and Δ*admX* (blue) in LB medium at 25 °C. A153 wt harbouring the empty reporter plasmid (black) was used as negative control in the assays. Data are the mean and standard deviation of three biological replicates.

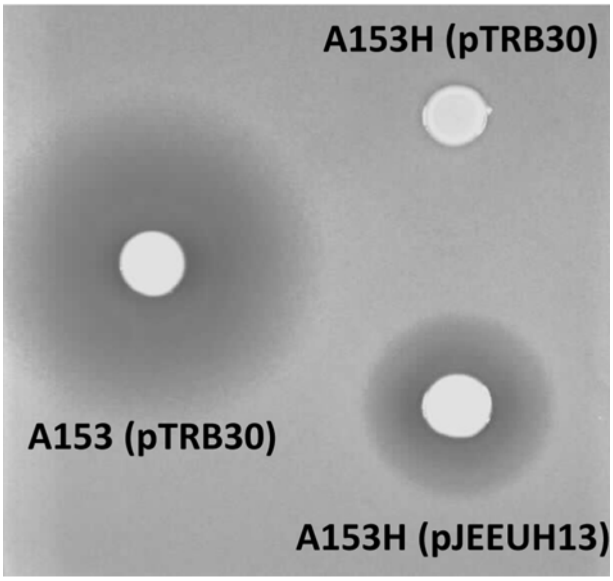

Supplementary Figure S8. Genetic complementation of *Serratia plymuthica* A153 strain A153H. Expression of *hfq in trans* in A153 Δ*hfq* restored andrimid production and therefore the antibacterial activity against *Bacillus subtilis*. Induction of Hfq expression was done by addition of 0.1 mM of IPTG. The bioassays were repeated at least three times, and a representative figure is shown. Pictures were taken after 48 h of incubation at 25 °C.

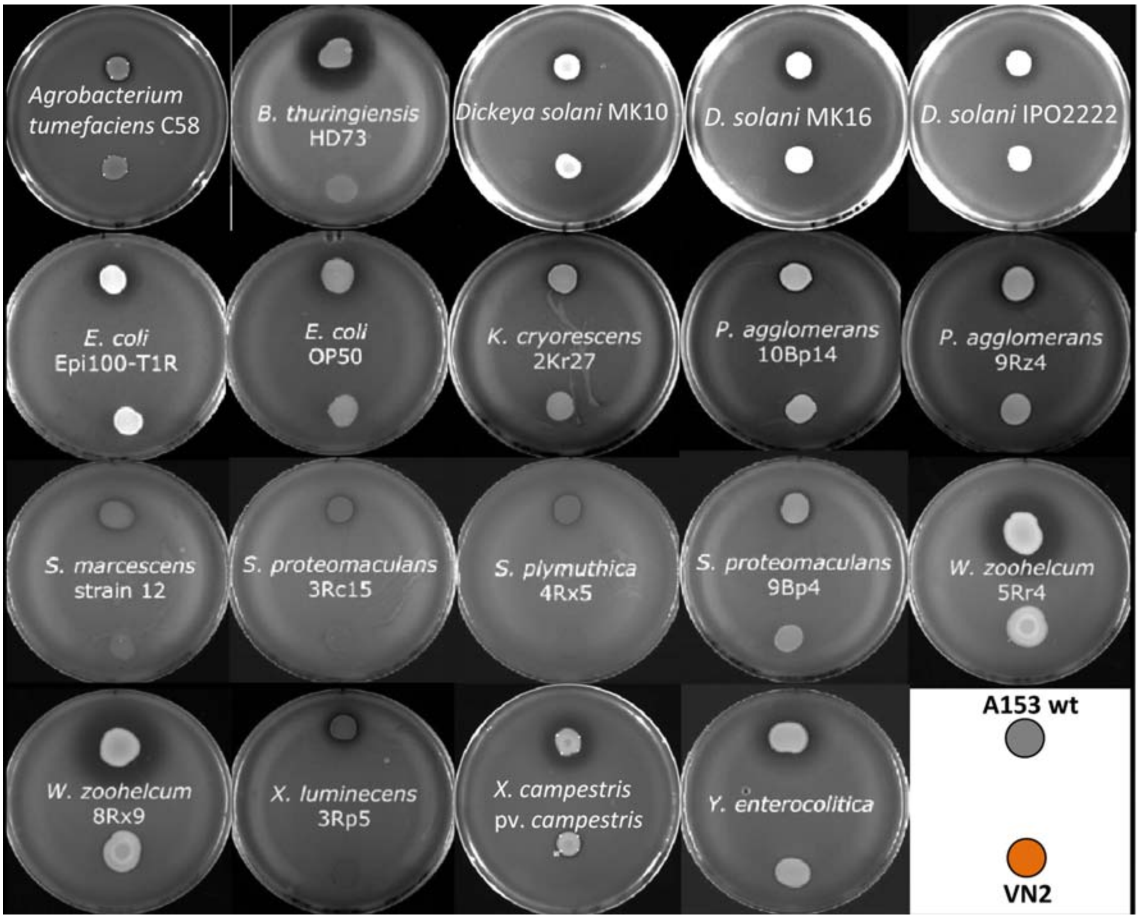

Supplementary Figure S9: Sensitivities of different bacterial strains to the antibiotic andrimid. Bioactivities of *Serratia plymuthica* A153 and the non-andrimid producing mutant of A153, VN2, against ecologically different bacterial strains. For the assays, an indicator top agar lawn was prepared as described in "Experimental procedures," and 5 µl overnight cultures of the A153 strains were spotted on the surface of the bacterial indicator agar lawns. The bioassays were repeated three times, and representative results are shown. Pictures were taken after 48 h of incubation at 25 °C. The strains used are described in Table 1 and supplementary Table S2.

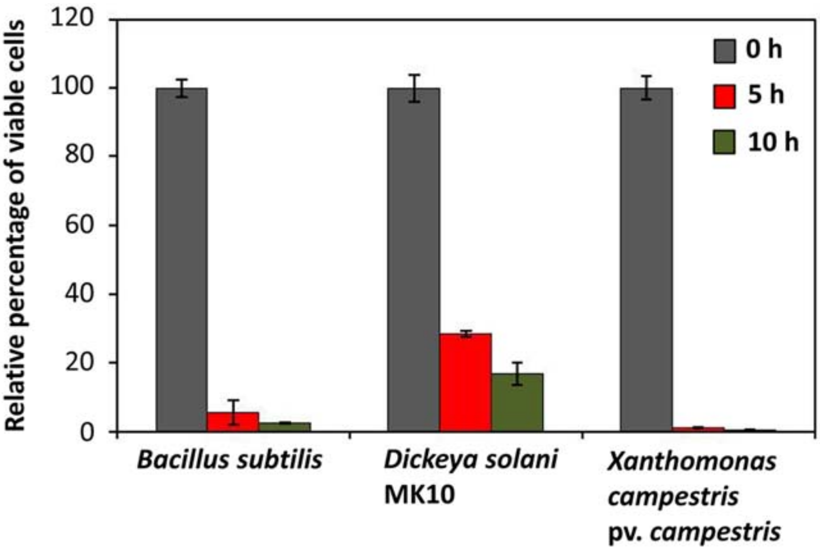

Supplementary Figure S10: Andrimid shows antibacterial activity against *Bacillus subtilis*, *Dickeya solani* and *Xanthomonas campestris* pv. *campestris*. Recovery of viable *Bacillus*, *Dickeya* and *Xanthomonas* cells grown in the presence of A153 JH6 (andrimid positive, zeamine negative) and A153 XJH6 (andrimid and zeamine negative) supernatants. The values showed the percentage of viable cells in the presence of JH6 supernatants relative to the number of viable cells in the presence of XJH6 supernatants. For the assays, overnight bacterial cultures of *Bacillus*, *Dickeya* and *Xanthomonas* were adjusted to an optical density at 600 nm (OD_{600}) of 0.1 and grown at 30 ºC with orbital shaking (225 rpm). At an OD_{600} of 0.4, 10 mL of the bacterial culture was removed and pelleted by centrifugation at 4,000 x *g* for 10 min at room temperature. The pellet was resuspended in 5 ml of 2X LB and 5 ml supernatants of an overnight culture of A153 JH6 or A153 XJH6 were added to the bacterial culture. Samples were taken after 5 and 10 h of incubation and the number of colony forming units (CFU) were determined. Data are the mean and standard deviation of three biological replicates.

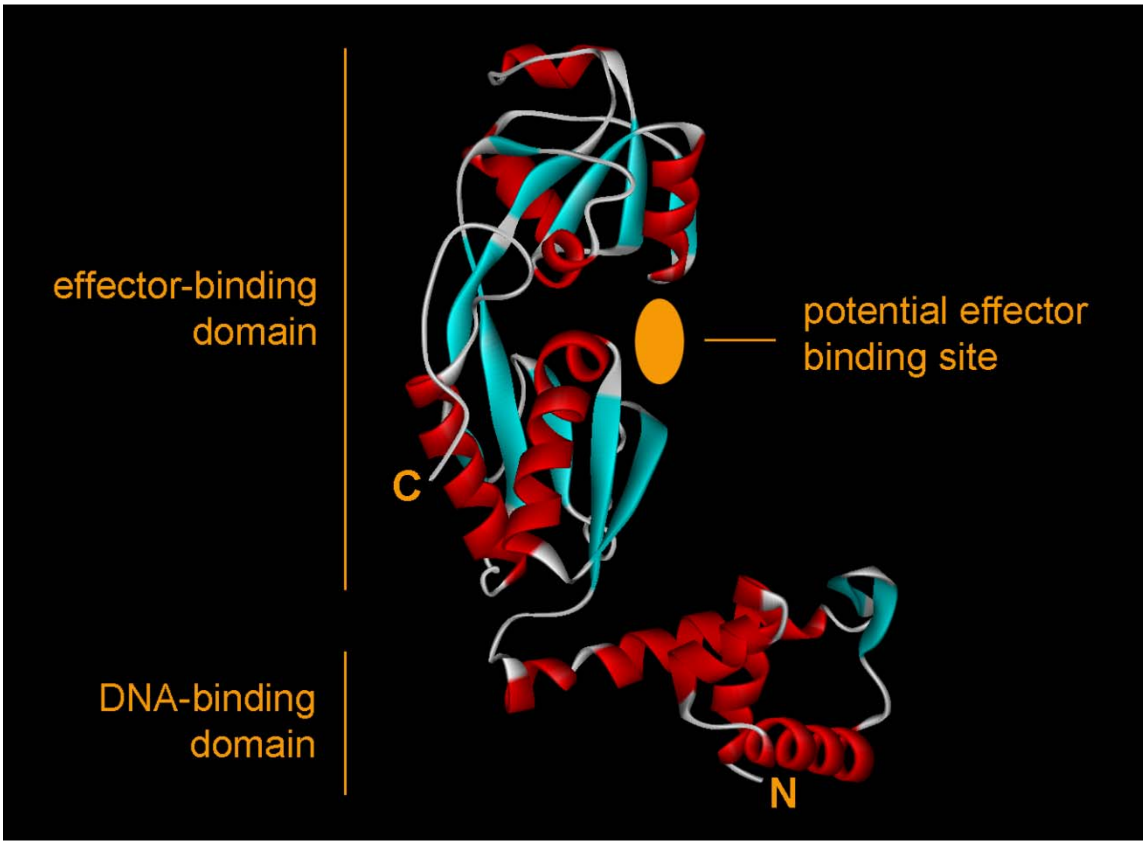

Supplementary Figure S11: Homology model of AdmX. The model was generated by the Geno3D modeling algorithm (Combet *et al*., 2000) and the structure of the BenM transcriptional regulator (PDB ID 3K1N) as template. The site for the binding of potential effector molecules is indicated.

REFERENCES

- Aucken, H.M., and Pitt, T.L. (1998) Antibiotic resistance and putative virulence factors of *Serratia marcescens* with respect to O and K serotypes. *J Med Microbiol* **47**: 1105-1113

- Berg, G., Roskot, N., Steidle, A., Eberl, L., Zock, A., and Smalla, K. (2002) Plant-dependent genotypic and phenotypic diversity of antagonistic rhizobacteria isolated from different *Verticillium* host plants. *Appl Environ Microbiol* **68:** 3328–3338.

- Brenner,S.(1974).The genetics of *Caenorhabditis elegans*. *Genetics* **77**: 71–94.

- Combet, C., Blanchet, C., Geourjon, C., and Deleage, G. (2000) NPS@: network protein sequence analysis. *Trends Biochem Sci* 25: 147-150.

- Fineran, P.C., Everson, L., Slater, H., and Salmond, G.P. (2005) A GntR family transcriptional regulator (PigT) controls gluconate-mediated repression and defines a new, independent pathway for regulation of the tripyrrole antibiotic, prodigiosin, in *Serratia*. *Microbiology* **151**: 3833-3345.

- Freiberg, C., Pohlmann, J., Nell, P.G., Endermann, R., Schuhmacher, J., Newton, B., *et al*., (2006) Novel bacterial acetyl coenzyme A carboxylase inhibitors with antibiotic efficacy *in vivo*. *Antimicrob Agents Chemother* **50**:2707-2712.

- Liu, G., Song, L., Shu, C., Wang, P., Deng, C., Peng, Q., Lereclus, D., Wang, X., Huang, D., Zhang, J., and Song, F. (2013) Complete genome sequence of *Bacillus thuringiensis* subsp. *kurstaki* strain HD73. *Genome Announc* **1**: e0008013.

- Matilla, M.A., Stöckmann, H., Leeper, F.J., and Salmond, G.P.C. (2012) Bacterial biosynthetic gene clusters encoding the anti-cancer haterumalide class of molecules: biogenesis of the broad spectrum antifungal and antioomycete compound, oocydin A. *J Biol Chem* **287:** 39125–39138.

- Pohlmann, J., Lampe, T., Shimada, M., Nell, P.G., Pernerstorfer, J., Svenstrup, N., *et al*., (2005) Pyrrolidinedione derivatives as antibacterial agents with a novel mode of action. *Bioorg Med Chem Lett* **15**:1189-1192.

- Pritchard, L., Humphris, S., Baeyen, S., Maes, M., Van Vaerenbergh, J., Elphinstone, J., Saddler, G., and Toth, I. (2013a). Draft genome sequences of four *Dickeya dianthicola* and four *Dickeya solani* strains. *Genome Announc* 1(4). pii: e00087-12.
- Spaink, H.P., Okker, R.J.H., Wijffelman, C.A., Pees, E., and Lugtenberg, B.J.J. (1987) Promoters in the nodulation region of the *Rhizobium leguminosarum* Sym plasmid pRL1JI*. Plant Mol Biol* 9: 27-39.
- Wood, D.W., Setubal, J.C., Kaul, R., Monks, D.E., Kitajima, J.P., Okura, V.K. *et al*. (2001) The genome of the natural genetic engineer *Agrobacterium tumefaciens* C58. *Science* **294**: 2317-23.
